# Quantifying force-field and partial-charge uncertainty in high-throughput grand canonical Monte Carlo screening of metal–organic frameworks for CO_2_ capture

**DOI:** 10.1039/d6ra09230f

**Published:** 2026-11-13

**Authors:** Mohammad Masrur Hossain

**Affiliations:** a Department of Industrial and Systems Engineering, The University of Tennessee Knoxville TN 37996 USA masrurhossain99@gmail.com

## Abstract

Grand-canonical Monte Carlo (GCMC) screens rank metal–organic frameworks (MOFs) for CO_2_ capture, but their force field and partial-charge scheme are chosen by convention. Here one such family of screens is analysed as a measurement system: in a gauge repeatability and reproducibility (R&R) study of the CRAFTED database, its rigid MOFs are the parts and its 12 generic force-field and charge-scheme protocols the appraisers (pure CO_2_ and N_2_, 273–323 K, 0.1–10 bar). At flue-gas conditions (CO_2_, 298 K, 0.1 bar) the measurement system, nearly all of it the protocol, accounts for 32% of the variance in predicted uptake across 632 MOFs (% GRR 56%, AIAG study variation; 42–62% with protocols resampled), and the signal-to-noise ratio is 2.1 (1.8–3.1): about two classes of material. The dominant error is material-specific interaction, not a global bias; charges derived from or trained on DFT lower % GRR only to 33%. Only three of one protocol's top-10 MOFs survive in another's. Protocol sensitivity is partly predictable from structure (*R*^2^ = 0.43): narrow pores, light metals and polar linkers fare worst, and metal-free covalent organic frameworks no better. In 330 new RASPA3 runs, replicates scatter half as much as the database's reported uncertainty for the same MOFs: the low resolution is protocol disagreement, not noise. CRAFTED's shifted-potential cut-off gives uptakes 20% below tail-corrected ones, which do not depend on the cut-off distance. Ten of 21 published uptakes of rigid frameworks lie outside the whole protocol range, nine below it. Such screens should report % GRR and NDC with their rankings.

## Abstract 2

GCMC screening treated as a measurement system: force-field and charge-scheme choice, with a little sampling noise, accounts for 32% of the variance in predicted CO_2_ uptake of 632 MOFs (% GRR 56%), so only about two classes of material are resolved.

## Graphical abstract

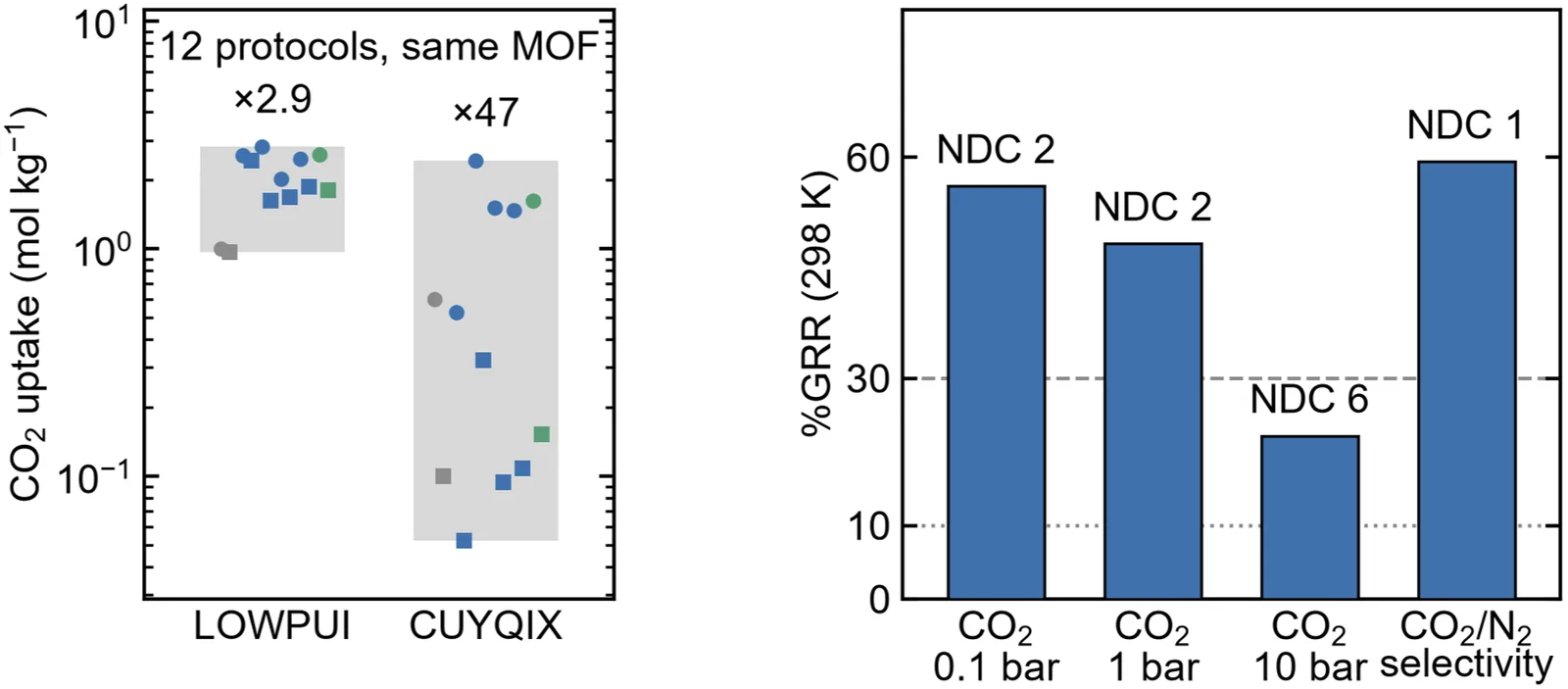

## 1 Introduction

Computational screening of nanoporous materials for CO_2_ capture rests on grand-canonical Monte Carlo (GCMC) simulations whose inputs—a generic force field such as UFF or DREIDING and a partial-charge scheme for the framework atoms—are chosen by convention rather than measured. Several studies have shown that these choices move predicted uptakes and process rankings substantially,^1–4^ and a companion study in preparation^5^ asks whether machine-learned design rules for CO_2_ uptake survive such changes; it shares no data, results, figures or tables with this article. What is missing is a standard way to state the resolution of such a screen: given the disagreement between reasonable protocols, how many materials can the simulation actually tell apart?

Manufacturing metrology has answered this question for physical gauges for four decades. A gauge repeatability and reproducibility (gauge R&R) study measures the same parts with several appraisers, decomposes the variance into part, appraiser, part × appraiser interaction and repeatability, and reports the share of the variation attributable to the measurement system (% GRR) and the number of distinct categories the gauge can resolve (NDC).^6,7^ The rules of thumb of the Automotive Industry Action Group (AIAG) call a gauge acceptable below 10% GRR, marginal up to 30% and unacceptable above, and require NDC ≥5 to sort parts. This framework transfers directly to simulated adsorption: MOFs are the parts, simulation protocols are the appraisers, and the Monte Carlo uncertainty is the repeatability.

The CRAFTED database^4,8^ makes the core of such a study possible without new simulations. It holds CO_2_ and N_2_ isotherms for the same 690 CoRE MOF 2014 structures^9,10^ and 667 CURATED COFs^11^ under all 12 combinations of two force fields and six charge schemes at three temperatures, with the Monte Carlo uncertainty of every point—a fully crossed, balanced design, exactly what a gauge study needs (Fig. 1). This paper (i) estimates the variance components and their confidence intervals for CO_2_ uptake, N_2_ uptake and CO_2_/N_2_ selectivity at three temperatures and pressures; (ii) translates them into the decision a screen makes, the overlap of top-*k* short-lists between protocols; (iii) models which structural features make a material protocol-sensitive; (iv) compares metal-containing MOFs with metal-free COFs; (v) reproduces the database with an independent code (RASPA3 (ref. 12)) and measures, in 330 new simulations of 30 MOFs, the factors CRAFTED holds fixed—replicate seed, sampling length, CO_2_ model and cut-off convention; and (vi) anchors the simulated spread to the public experimental isotherms of the database's materials.^13^ The result is both a finding about CO_2_ screening and a reporting standard that any simulated-property screen can adopt.

**Fig. 1:**
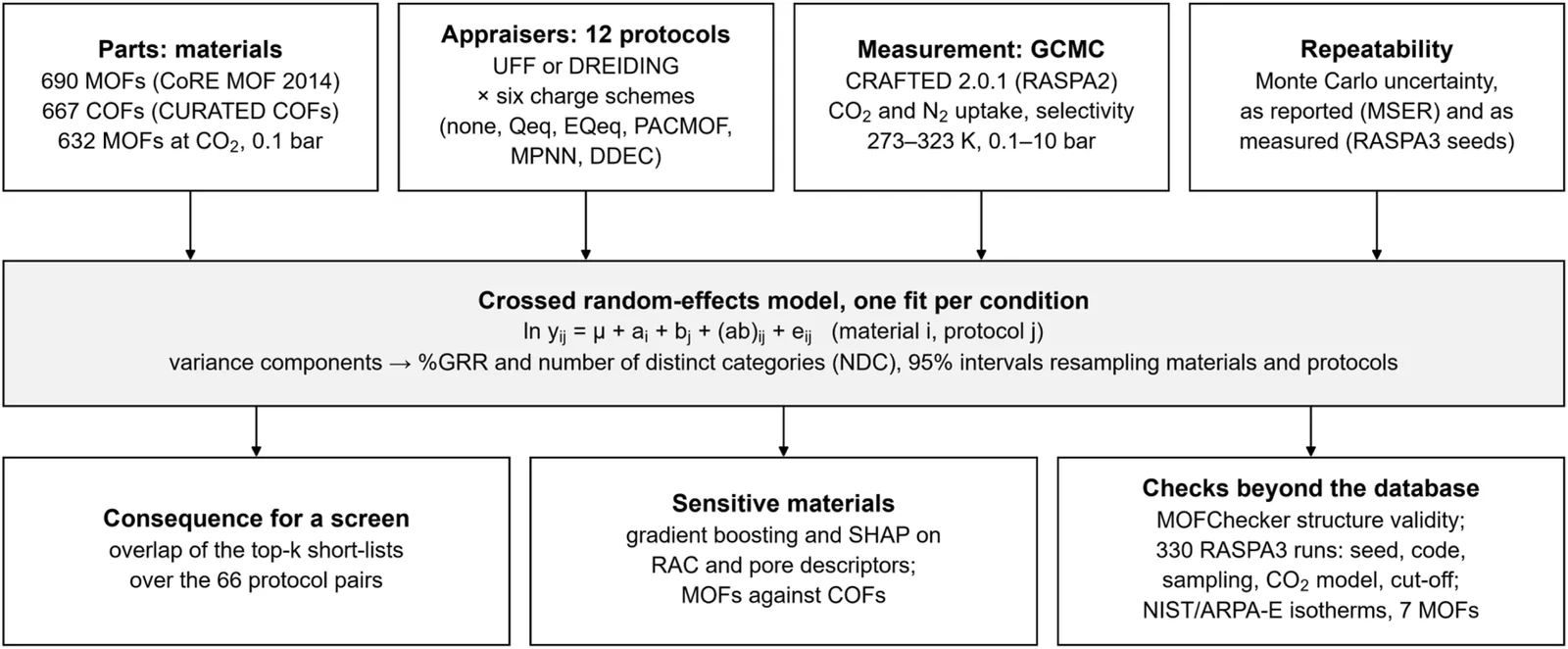
Design of the study: GCMC screening analysed as a measurement system. The parts of the gauge repeatability and reproducibility (R&R) study are the materials of CRAFTED 2.0.1 (632 MOFs used at 0.1 bar; the 667 COFs are analysed in the same way), the appraisers are the 12 simulation protocols (UFF or DREIDING force field combined with six partial-charge schemes), the measurement is the GCMC uptake at 273, 298 and 323 K, and the repeatability is the Monte Carlo uncertainty, as reported by the database and as measured with independent RASPA3 runs. A crossed random-effects model, fitted for each condition, splits the variance into material, protocol, material × protocol interaction and repeatability and gives % GRR, the share of the observed variation caused by the measurement system, and NDC, the number of classes of materials the simulation can tell apart. The bottom row lists the analyses built on the decomposition.

## 2 Methods

### 2.1 Data

CRAFTED 2.0.1 (ref. 4 and 8) contains 690 MOFs from CoRE MOF 2014 and 667 COFs from CURATED COFs, each simulated with UFF^14^ and DREIDING^15^ combined with six charge schemes (no charges; Qeq,^16^ which in CRAFTED is the default charge equilibration of RASPA 2.0.45 and assigns one charge per element; EQeq;^17^ PACMOF;^18^ MPNN;^19^ and DDEC^10^) at 273, 298 and 323 K in RASPA2 (ref. 20) with the TraPPE models of CO_2_ and N_2_,^21^ at 13 pressures from 100 Pa to 10 bar, with 10 000 Monte Carlo cycles per point, MSER truncation^22,23^ and the uptake reported as mean ± uncorrelated standard deviation. ln(uptake) was analysed at 0.1, 1 and 10 bar for CO_2_ and N_2_, together with the selectivity proxy *S* = [*q*(CO_2_, 0.2 bar)/0.2 bar]/[*q*(N_2_, 1 bar)/1 bar], the ratio of pure-gas uptakes per unit pressure at the tabulated pressures nearest the 0.15 and 0.85 bar partial pressures of a dry flue gas. Materials adsorbing less than 0.05 mol kg^−1^ under any protocol at a condition were excluded from that condition, because the logarithm of a noise-level uptake is meaningless; only complete 12-protocol blocks were kept. Any threshold between 0.005 and 0.05 mol kg^−1^ gives % GRR within 0.4 percentage points at CO_2_, 298 K and 0.1 bar (Table S1 (SI)); of the 58 MOFs that 0.05 mol kg^−1^ removes there, 19 are removed only because of the two no-charge protocols. Physically implausible uptakes were flagged but kept: a value at 0.1 or 1 bar larger than the 10 bar uptake of the same MOF under every other charge scheme is flagged (Table S2 (SI)), and the main results are repeated without the flagged MOFs.

### 2.2 Measurement-systems model

For each condition, ln *y*_*ij*_ = *µ* + *a*_*i*_ + *b*_*j*_ + (*ab*)_*ij*_ + *e*_*ij*_, with material *i* and protocol *j* as crossed random effects, their interaction (*ab*)_*ij*_ and the Monte Carlo error *e*_*ij*_. With one simulation per cell, interaction and repeatability are separated through the reported uncertainty: the repeatability variance is the median squared relative error—the median rather than the mean, because a minority of cells, mostly at low uptake, have very large relative errors (with the mean, the interaction sd at CO_2_, 298 K and 0.1 bar falls from 0.460 to 0.389, while % GRR and NDC do not change; Table S3 (SI))—and the interaction variance is the residual mean square minus that quantity, clipped at zero. The components were estimated by the method of moments and cross-checked by restricted maximum likelihood (statsmodels^24^ MixedLM), which agrees to three decimals (Table S4 (SI)). Following the AIAG manual,^6,7^ % GRR = 100 √[(*σ*_protocol_^2^ + *σ*_interaction_^2^ + *σ*_MC_^2^)/*σ*_total_^2^] and NDC = ⌊1.41*σ*_material_/*σ*_GRR_⌋. Two AIAG percentages are in use and they differ: % GRR as quoted here is the study variation, a ratio of standard deviations, and its square is the contribution to the total variance—at CO_2_, 298 K and 0.1 bar 56.1% of the study variation is 31.5% of the variance. Both are tabulated for every condition in Table S5 (SI). NDC, the signal-to-noise ratio 1.41 *σ*_material_/*σ*_GRR_, is reported before rounding and with its bootstrap interval as well as in the floored form of the manual. Confidence intervals (95%) come from a cluster bootstrap over materials with 500 resamples, because the materials, not the individual simulations, are the sampling units. The protocols are a sample of the protocols in use as well, so the intervals of the headline conditions are also given with the protocols resampled: a two-way (pigeonhole) bootstrap of materials and protocols,^25^ a bootstrap of materials and of the six charge schemes, each drawn with both force fields, and a leave-one-protocol-out jackknife (1000 resamples; Table S6 (SI)).

### 2.3 Decision consequence

For every condition the materials were ranked under each protocol. For *k* = 10, 25, 50 and 100 the mean pairwise Jaccard overlap |*A*∩*B*|/|*A*∪*B*| of the top-*k* sets over the 66 protocol pairs was computed, together with the number of the reference (UFF + DDEC) top-*k* retained under each other protocol, the number of materials in the top-*k* of all 12 protocols and the mean pairwise Spearman correlation of the full rankings.

### 2.4 Protocol-sensitivity model

The sensitivity of a material is the standard deviation of its ln(uptake) across the 12 protocols after the protocol main effects are removed, that is, its share of the interaction; the charge-scheme spread within UFF and the UFF–DREIDING gap were analysed as well. Gradient-boosted trees (XGBoost;^26^ 400 trees, depth 4, learning rate 0.03) were trained on the 156 revised autocorrelation (RAC)^27,28^ and 14 Zeo++^29^ pore descriptors supplied with CRAFTED, evaluated by five-fold cross-validation and explained with TreeSHAP;^30^ the direction of each descriptor's effect was read from the mean SHAP value of the MOFs in its lowest and highest quintiles, because several effects are not monotonic.

### 2.5 What the gauge framing assumes

A gauge study of a simulation differs from one of a physical instrument in three ways, and the conclusions are drawn accordingly. There is no reference value: % GRR and NDC describe precision across protocols, not accuracy, which is why Section 3.9 anchors the spread to measured isotherms. The appraisers are not interchangeable operators but different physical models, so the protocol variance is a property of the family of protocols in common use; the uncertainty that comes from having only twelve of them is carried by the two-way intervals of Section 2.2, and the subsets in Table S7 (SI) show how the variance changes when the family is narrowed. And there is one simulation per cell, so repeatability comes from the uncertainty the database reports and, in Section 3.8, from replicate runs. The variance decomposition itself assumes only the crossed random-effects model above; the AIAG thresholds (% GRR <10% acceptable, 10–30% marginal, >30% unacceptable, NDC ≥5 to sort parts) are a convention from manufacturing^6^ and are used here as a common scale, not as a law of simulation. Because NDC compares the spread of the materials with the spread of the measurement system, it depends on the population of materials as well as on the protocols; it is therefore reported for the whole database and for the subsets a screen actually works with (Table S8 (SI)).

### 2.6 Structural validity

The 690 MOF structures were screened with MOFChecker^31^ (mofchecker-next 0.1.2) for atomic overlaps, over- and under-coordinated C, N and H atoms, under-coordinated rare-earth and alkali metals, geometrically exposed metals, suspicious terminal oxo groups, isolated molecules and missing hydrogens; open metal sites and high charges were treated as properties, not errors. The variance decomposition was repeated on the clean and on the flagged subsets (200 bootstrap resamples).

### 2.7 Cross-code reproduction

One complete isotherm (MULQOA, UFF + DDEC, 298 K) was re-simulated with RASPA3 3.1 (ref. 12) using CRAFTED's CIF, force-field parameters and cell replication, 10 000 cycles per pressure and pyMSER truncation,^23^ and compared point by point with the database through *z* = (difference)/(combined uncertainty). The simulation settings are listed in SI Section S2.

### 2.8 Factors CRAFTED lacks

Thirty MOFs were drawn from the sensitivity ranking of Section 2.4—the 10 most and the 10 least protocol-sensitive and 10 at random from the middle quartiles—and re-simulated with RASPA3 under the reference protocol (UFF + DDEC, 298 K) in a one-factor-at-a-time design of 240 runs: (A) two independent random seeds for CO_2_ at 0.1 and 1 bar, which give the run-to-run repeatability, sd = √(〈(Δ ln *u*)^2^〉/2), and, averaged, the code effect against the database; (B) N_2_ at 1 bar with 10 000 and 40 000 cycles; (C) the EPM2 CO_2_ model^32^ instead of TraPPE^21^ at 0.1 bar; (D) a 12.0 Å truncated cut-off with tail corrections instead of CRAFTED's 12.8 Å shifted potential at 0.1 bar. Factor (D) moves the cut-off distance and the treatment of the potential beyond it at the same time, so it was completed into a 2 × 2 design on the same MOFs with 90 further runs: 12.0 Å and 12.8 Å, each with the shifted potential and with a truncated potential plus analytic tail corrections, together with 14.0 Å shifted to give the distance a direction. The distance effect is then the change at constant treatment and the treatment effect the change at constant distance, each read at both levels of the other setting; the difference between the two readings is their interaction. For each factor the mean change of ln(uptake), its standard deviation across MOFs (the material-specific part) and the number of MOFs whose change lies inside the combined Monte Carlo noise (2*σ*) are compared with the CRAFTED variance components; because the sample over-represents the extremes of sensitivity, each factor is also reported for the three groups separately (Table S9 (SI)). Conditions that disagreed with the database by more than 2*σ* were re-run for 40 000 cycles with the per-cycle trace saved, together with two well-behaved controls per pressure. Finally, the decomposition of Section 2.2 was repeated with the measured repeatability in place of the reported uncertainty, the value measured under UFF + DDEC standing for all 12 protocols, and the reported uncertainty was compared with the measured one like for like—the same 30 MOFs under the same protocol (Table S10 (SI)); for N_2_ the repeatability was inferred from the 10 000- *versus* 40 000-cycle difference (sd/√1.25), and for selectivity the CO_2_ and N_2_ values were combined in quadrature.

### 2.9 Experimental anchor

The chemical formula of every CRAFTED CIF was matched against those of well-known MOFs, and each match was searched by name in the NIST/ARPA-E database of adsorption isotherms.^13^ Every experimental pure-CO_2_ isotherm at 296–300 K of a matched material was converted to mol kg^−1^ and bar (volumetric units through the crystal density of the CIF); the uptake at 0.1, 1 and 10 bar was interpolated on log–log axes when bracketed by measured points and compared with the 12 CRAFTED protocol values at the same temperature. The comparison was then widened to the other two temperatures of the database, taking every experimental isotherm within 3 K of 273 K and of 323 K as well (no temperature correction is applied, so the window is kept tight), and the spread between independent papers that measured the same material at the same condition was recorded as the experimental counterpart of the protocol spread. Duplicate entries of the same curve were removed. Where CoRE MOF 2014 holds several CIFs of the same composition, the spread of the reference-protocol uptake across those CIFs was recorded as a structure-source effect. The values outside the protocol range were counted by material, with how far outside and on which side (Table S11 (SI)), and the spread between laboratories was compared with that between protocols as a standard deviation as well as a range.

### 2.10 Software and reproducibility

All analyses were run in Python 3.12.5 with pandas, NumPy, statsmodels, scikit-learn, XGBoost, SHAP and matplotlib (versions in Table S12 (SI)) on a laptop (Windows, 16 logical cores); the 330 RASPA3 simulations took about 160 CPU-hours and every other step runs in minutes. Every number, table and figure in this article is regenerated from the public database by the deposited code,^33^ with one command per step (SI Section S1).

## 3 Results

### 3.1 Variance components

Table 1 and Fig. 2 give the decomposition at 298 K; all temperatures and pressures are listed in Table S13 (SI). For CO_2_, % GRR is 56.1% (95% CI 53.4–58.8%) at 0.1 bar (NDC 2), 48.3% (95% CI 46.0–50.9%) at 1 bar (NDC 2) and 22.2% (95% CI 19.1–25.2%) at 10 bar (NDC 6). These intervals resample the materials; with the protocols resampled as well they are 41.5–62.5%, 36.0–53.6% and 16.3–25.5% (two-way bootstrap; Table S6 (SI)), and leaving out one protocol at a time moves % GRR at 0.1 bar between 52.1% and 57.4%. Every interval at 0.1 and 1 bar stays above the AIAG limit of 30%, and the 10 bar interval inside the marginal band. At 0.1 bar the material × protocol interaction (sd 0.460 in ln units) exceeds the protocol main effect (0.328) and the reported Monte Carlo term (0.157); at 10 bar all three shrink and the material term (0.717) dominates. The pattern is the same at 273 and 323 K: % GRR at 0.1 bar is 54.6% at 273 K and 60.8% at 323 K, but on the 553 MOFs that pass the threshold at all three temperatures it is 60.1%, 60.4% and 60.8%; the apparent rise with temperature comes from the weakly adsorbing MOFs that the threshold removes at the higher temperatures (Table S14 (SI)). N_2_ at 1 bar gives 49.3% (95% CI 47.0–51.6%), and CO_2_/N_2_ selectivity gives 60.5%, 59.4% and 62.5% at 273, 298 and 323 K, with NDC = 1 throughout. For N_2_ at 0.1 bar, N_2_ at 1 bar and CO_2_/N_2_ selectivity the interaction estimate is zero and the reported Monte Carlo term (0.29–0.65) dominates; Section 3.8 shows that this is an artefact of the reported uncertainty, which overstates the true run-to-run scatter, and that the interaction reappears once the measured repeatability is used.

**Table 1:** Variance components (standard deviations in ln units), % GRR with bootstrap 95% confidence interval (materials resampled; with the protocols resampled as well in Table S6 (SI)) and number of distinct categories (NDC) for MOFs and COFs at 298 K; *n* is the number of materials with a complete 12-protocol block. All temperatures and pressures are listed in Table S13 (SI)

Class	Response	*P*/bar	*n*	sd material	sd protocol	sd interaction	sd MC	% GRR [95% CI]	NDC
MOF	CO_2_	0.1	632	0.866	0.328	0.460	0.157	56.1 [53.4, 58.8]	2
MOF	CO_2_	1	669	0.614	0.176	0.284	0.053	48.3 [46.0, 50.9]	2
MOF	CO_2_	10	677	0.717	0.071	0.145	0.024	22.2 [19.1, 25.2]	6
MOF	N_2_	1	614	0.546	0.107	0.000	0.290	49.3 [47.0, 51.6]	2
MOF	CO_2_/N_2_ selectivity		613	0.498	0.188	0.000	0.316	59.4 [57.3, 62.5]	1
COF	CO_2_	0.1	334	0.865	0.387	0.000	0.508	59.4 [55.7, 63.5]	1
COF	CO_2_	1	663	0.678	0.342	0.303	0.220	60.0 [57.0, 62.9]	1
COF	CO_2_	10	663	0.455	0.205	0.159	0.072	50.9 [47.7, 54.1]	2
COF	N_2_	1	655	0.458	0.166	0.000	0.592	80.2 [78.0, 82.2]	1
COF	CO_2_/N_2_ selectivity		629	0.625	0.263	0.000	0.803	80.4 [78.4, 82.3]	1

**Fig. 2:**
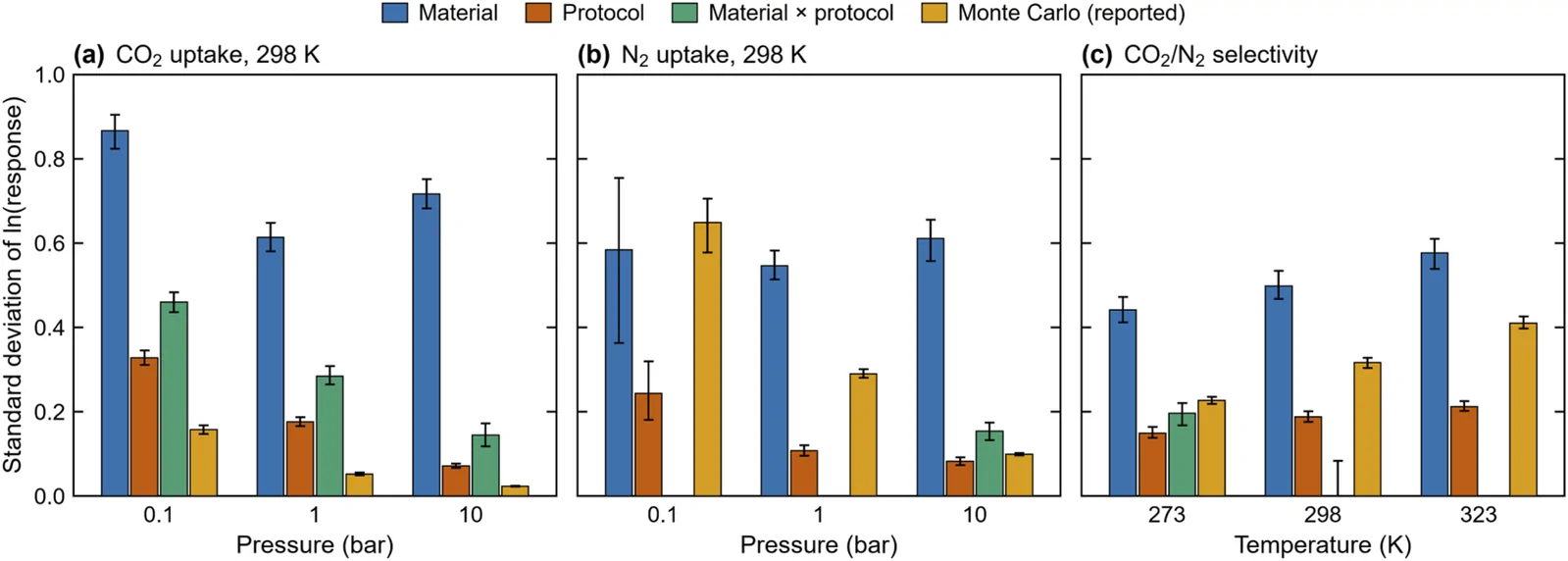
Variance components of the simulated adsorption of the MOFs. Standard deviation (ln units) of the material, protocol, material × protocol and reported Monte Carlo components for (a) CO_2_ and (b) N_2_ uptake at 298 K and 0.1, 1 and 10 bar and (c) CO_2_/N_2_ selectivity at 273, 298 and 323 K (CRAFTED 2.0.1). Whiskers: 95% confidence intervals from a cluster bootstrap over the materials (500 resamples), which holds the 12 protocols fixed; with the protocols resampled as well the intervals are wider, most for the protocol component (Table S17 (SI)). A missing interaction bar was clipped at zero because the reported Monte Carlo term exceeds the residual scatter (a whisker without a bar is the bootstrap interval of such a component); Fig. 13 repeats the analysis with the measured repeatability. % GRR and NDC of each condition are listed in Table 1.

Two readings of the same decomposition are worth separating, because both are in use. The 56.1% quoted above is the AIAG study variation, a ratio of standard deviations; as a share of the variance the measurement system is 31.5% at 0.1 bar, 23.3% at 1 bar and 4.9% at 10 bar (Table S5 (SI)); at 0.1 bar the reported Monte Carlo term is 2.3 percentage points of it and the protocol, through its main effect and its interaction with the material, the other 29.2 (Table S15 (SI)). The number of distinguishable classes is 2.08 before rounding, with a bootstrap interval of 1.95–2.24 over the materials and of 1.76–3.09 with the protocols resampled as well, so the floor of two is not an artefact of a borderline estimate. It is, however, a property of this population of materials as much as of the protocols: on the middle half of the database by uptake the ratio falls to 0.85, and on the top decile—the short-list a screen would carry forward—to 0.41, while within the least protocol-sensitive third of the materials it reaches 3.15 (Table S8 (SI)). Resolution is therefore worst where a screen needs it, though partly by construction: in the top decile the material spread is only a fifth of the database's, so even a gauge that resolved five classes on the whole database would resolve about one there; the top-*k* overlap of Section 3.3 measures the short-list more directly.

The same decomposition on subsets of the protocol family shows where the disagreement comes from (Table S7 (SI); the materials are held fixed, so only the protocol set changes). Dropping the two protocols that carry no atomic charges lowers % GRR at 0.1 bar from 56.1% to 47.4% and leaves NDC at 2: the no-charge protocols inflate the protocol main effect (sd 0.328 → 0.155) but barely touch the material-specific interaction (0.460 → 0.438), which is the term that bounds the resolution. Restricting the family further to the charge schemes derived from or trained on DFT (DDEC, PACMOF, MPNN) gives % GRR 32.8% and NDC 4 at 0.1 bar and 31.7% at 1 bar—a real improvement on 56.1%, and still outside the AIAG acceptance band and short of NDC 5. Part of the improvement is built in: PACMOF and MPNN are machine-learned models trained to reproduce DDEC charges^18,19^—MPNN on the very DDEC charges that CRAFTED uses, computed for the CoRE MOF 2014 structures,^10^ so that its agreement with DDEC is largely in-sample—and the three schemes are approximations of one target rather than independent physical models. Against the measured isotherms of Section 3.9 the narrower family is not clearly better—its median is closer to the measurements of the rigid frameworks (median |ln(measured/simulated)| 0.26 against 0.34), but its range contains the measurement for only four of 21 values, against 11 for all 12 protocols (Table S16 (SI)). Using one force field with all six charge schemes (52.8% for UFF, 52.5% for DREIDING) does not help, because the charge scheme, not the force field, carries most of the material-specific disagreement.

### 3.2 Protocol biases

The protocol effects are known only as well as the protocols are sampled: at 0.1 bar the sd of the protocol main effect, 0.328, has a 95% interval of 0.311–0.346 with the materials resampled (Fig. 2) but 0.132–0.431 with the protocols resampled as well (Table S17 (SI)), so the biases below describe these twelve protocols. Relative to the DFT-charge reference (UFF + DDEC), omitting the charges multiplies the CO_2_ uptake at 0.1 bar by ×0.47 (UFF) and ×0.39 (DREIDING) on average; DREIDING with DDEC charges gives ×0.79; the four approximate charge schemes with UFF give ×1.13 (Qeq), ×1.03 (EQeq), ×0.99 (PACMOF) and ×1.04 (MPNN) (Fig. 3; all conditions in Table S18 (SI)). Every bias shrinks with pressure as the pores fill. The average biases of the charge schemes are small; the interaction term shows that their material-specific errors are not.

**Fig. 3:**
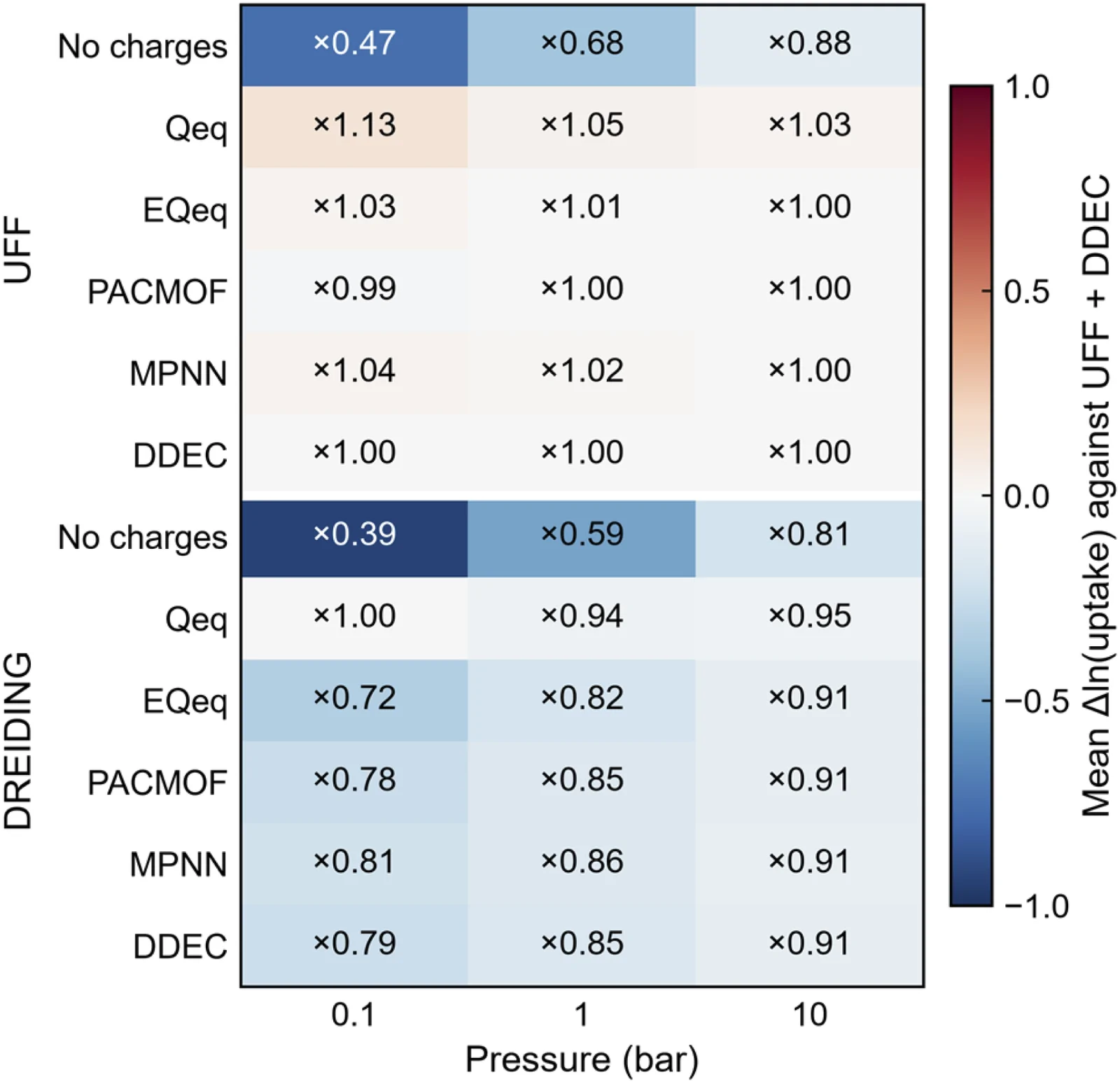
Mean bias of each protocol relative to UFF + DDEC. Mean change of ln(CO_2_ uptake) over the MOFs at 298 K and 0.1, 1 and 10 bar for each of the 12 protocols, relative to the reference protocol UFF + DDEC; each cell gives the corresponding factor on the uptake (blue: below the reference, red: above). The white line separates the UFF and DREIDING blocks.

### 3.3 Decision consequence

At 0.1 bar and 298 K the mean pairwise Jaccard overlap of two protocols' top-10 MOFs is 0.17 (0.24, 0.30 and 0.40 for the top-25, top-50 and top-100): on average only 3.0 of the reference top-10 remain in the top-10 of another protocol, and none under UFF + no charges (Table 2 and Fig. 4). No MOF is in the top-10 of all twelve protocols and only 10 are in every top-100 (mean Spearman rank correlation 0.76). At 10 bar the rankings converge (Spearman 0.98; top-100 Jaccard overlap 0.83, with 71 MOFs in every top-100). For selectivity the top-10 overlap is 0.29 and no MOF is in the top-10 of every protocol. All conditions are listed in Table S19 (SI).

**Table 2:** Agreement of the top-*k* short-lists of the 12 protocols for MOFs at 298 K: mean pairwise Jaccard overlap (66 protocol pairs), number of the reference (UFF + DDEC) top-*k* that remain in another protocol's top-*k* (mean over the other 11 protocols, minimum in parentheses), number of MOFs in the top-*k* of all 12 protocols and mean Spearman rank correlation. All conditions are listed in Table S19 (SI)

Response	*P*/bar	*k*	Mean Jaccard	Reference top-*k* kept, mean (min)	In all 12 top-*k*	Mean Spearman
CO_2_	0.1	10	0.17	3.0 (0)	0	0.76
CO_2_	0.1	100	0.40	62.0 (40)	10	0.76
CO_2_	1	10	0.29	4.9 (1)	0	0.86
CO_2_	1	100	0.53	74.0 (57)	30	0.86
CO_2_	10	10	0.41	6.5 (5)	1	0.98
CO_2_	10	100	0.83	91.3 (87)	71	0.98
CO_2_/N_2_ selectivity		10	0.29	5.0 (1)	0	0.69
CO_2_/N_2_ selectivity		100	0.42	64.2 (37)	11	0.69

**Fig. 4:**
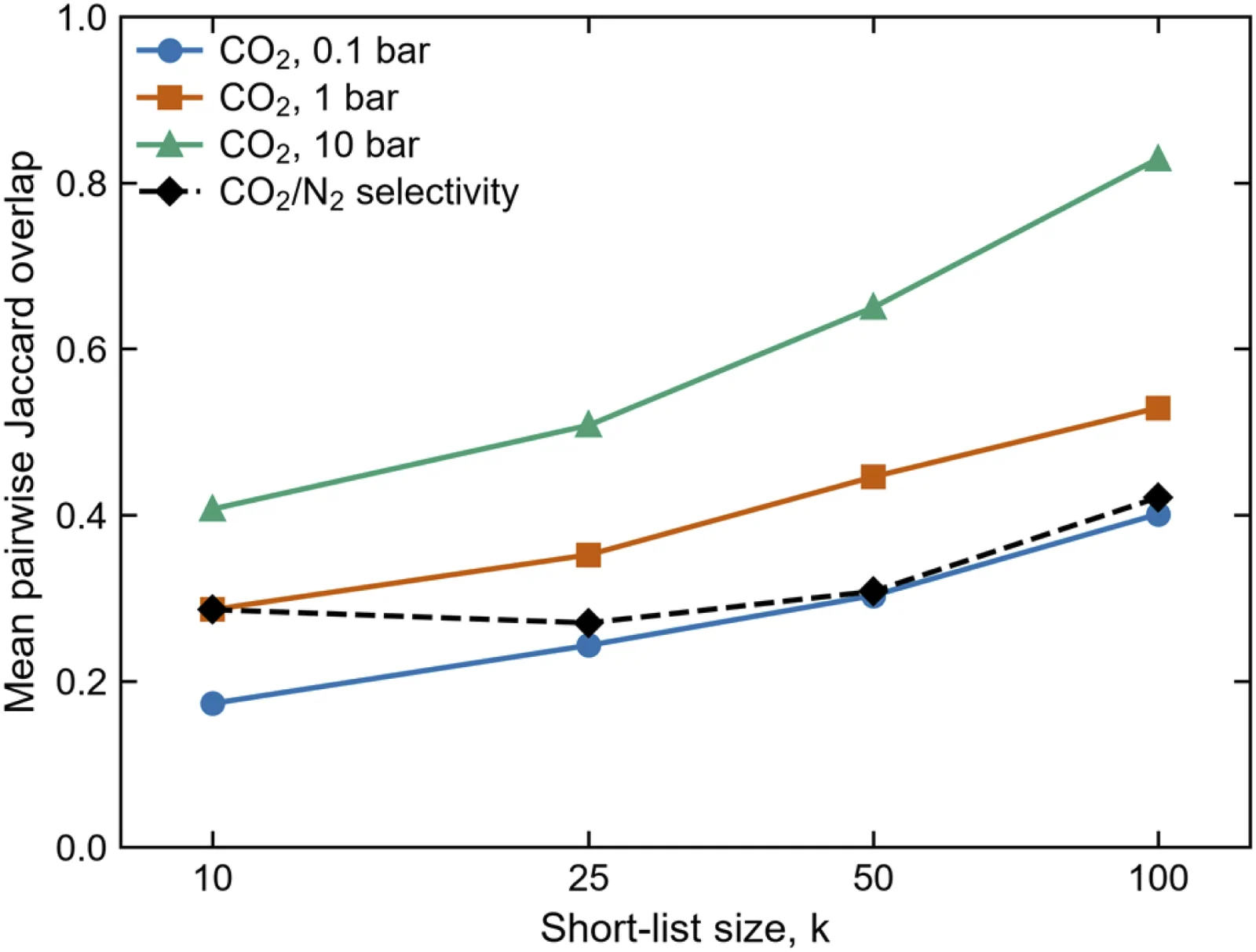
Agreement of the top-*k* short-lists between protocols. Mean pairwise Jaccard overlap |*A*∩*B*|/|*A*∪*B*| of the top-*k* MOFs over the 66 protocol pairs, for *k* = 10, 25, 50 and 100, with the MOFs ranked by CO_2_ uptake at 298 K and 0.1, 1 or 10 bar or by CO_2_/N_2_ selectivity. The values for *k* = 10 and 100 are listed in Table 2.

### 3.4 What makes a material protocol-sensitive

Seen from the material's side, the 12 protocols return uptakes that span a factor of 4.3 (median ln(max/min) = 1.47) for a typical MOF and more than a factor of 17 for one MOF in ten (Fig. 5); the direction of a protocol change differs between frameworks, which is what the interaction term measures. The per-MOF sensitivity has median 0.35 and 90th percentile 0.73 ln units (Table S20 (SI)); the most sensitive frameworks are LURGEL (1.53), CUYQIX (1.25), XINWUO (1.17), SENWIT (1.16) and PARHAS (1.15). LURGEL's place comes from the two Qeq protocols, which give it 25.3 and 27.3 mol kg^−1^ at 0.1 bar against 0.19–0.71 mol kg^−1^ under the other ten, and more than any other charge scheme gives it even at 10 bar. Values of this kind fail the plausibility screen of Section 2.1, which flags 52 values in 30 MOFs at 298 K and 0.1 bar, 51 of them under Qeq (Table S2 (SI)). The flagged MOFs include six of the 20 most sensitive ones but hardly move the gauge: without them % GRR at 0.1 bar is 55.1% (NDC 2.14), and without the two Qeq protocols 54.4% (Table S21 (SI)). After the screen the most sensitive frameworks are CUYQIX (1.25), XINWUO (1.17), SENWIT (1.16), PARHAS (1.15) and SENWEP (1.08). Gradient boosting predicts the sensitivity with a cross-validated *R*^2^ of 0.43, the charge-scheme spread with 0.48 and the force-field gap with 0.50 (Table S22 (SI)). The leading descriptors by mean |SHAP| (Fig. 6 and Table S23 (SI)) are, with the values that raise the predicted sensitivity in parentheses, the atomic number difference around the metal centre (depth 1; low values), the largest included sphere (low), the coordination number around the functional group (depth 2; low), the unit-cell volume (low) and the atomic number around the metal centre (depth 1; low). In chemical terms, light metal centres with light neighbours, the narrowest pores and polarizable, electronegative atoms around the linker connection points mark the protocol-sensitive frameworks—tight, electrostatics-dominated binding environments in which the charge scheme and the dispersion parameters matter most. Several effects are not monotonic (the included-sphere effect, for example, is confined to the narrowest fifth of the pores), which is why the direction is read from the extreme quintiles of each descriptor. Refitted without the two Qeq protocols, the model predicts the sensitivity with a cross-validated *R*^2^ of 0.48 (against 0.43 for the same code with all twelve), and its five leading descriptors are the atomic number difference around the metal centre (depth 1), the electronegativity around the metal centre (depth 1), the largest included sphere, the electronegativity around the linker-connecting atom (depth 3) and the electronegativity around the metal centre (depth 3); the metal centre and the pore size lead in both models, so the reading above does not rest on the Qeq values (Table S24 (SI)).

**Fig. 5:**
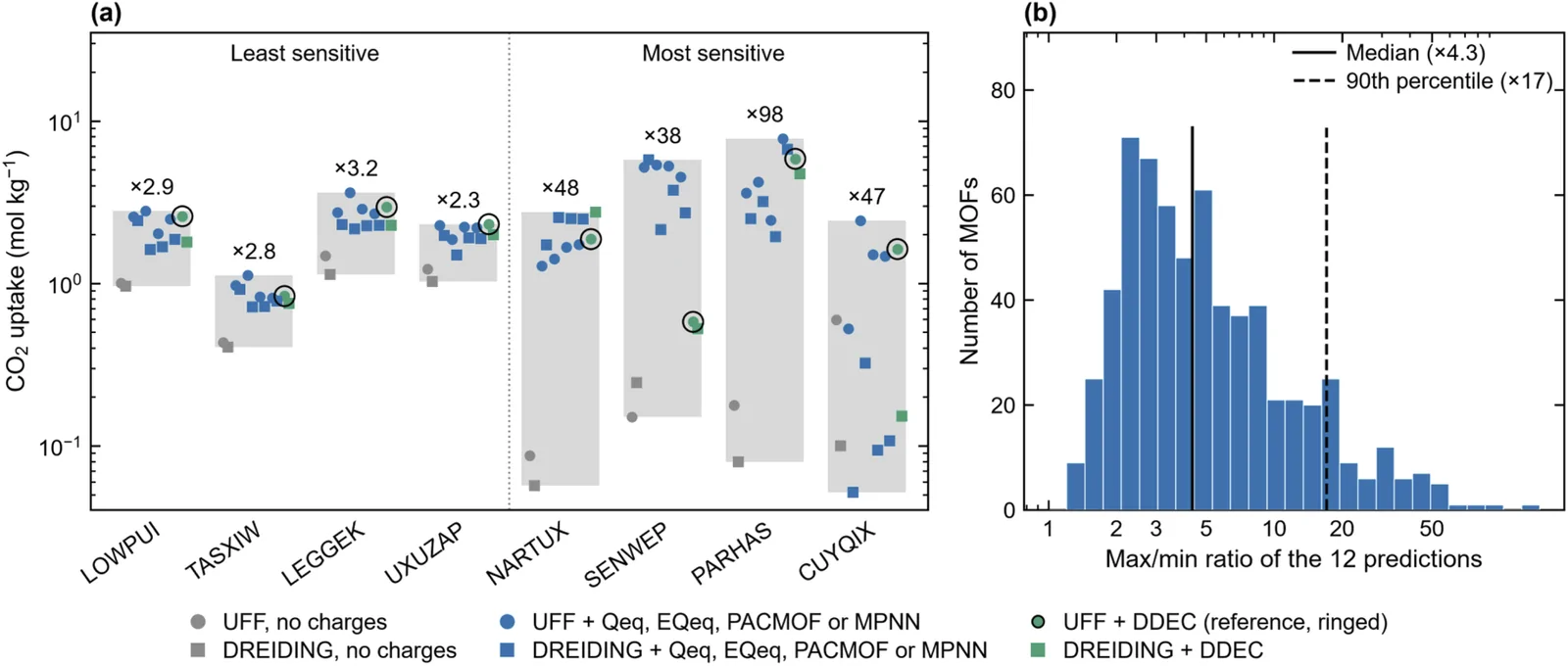
Predictions of the 12 protocols for individual MOFs. (a) CO_2_ uptake at 298 K and 0.1 bar under each protocol for the four least and the four most protocol-sensitive MOFs among those adsorbing at least 0.3 mol kg^−1^ under the reference protocol and not flagged by the plausibility screen of Section 2.1: circles UFF, squares DREIDING; grey no charges, blue Qeq, EQeq, PACMOF or MPNN charges, green DDEC charges; the reference protocol (UFF + DDEC) is ringed, the grey bar spans the twelve values and the label gives their max/min ratio. (b) Distribution of that ratio over all 632 MOFs (median ×4.3, 90th percentile ×17; 67% of the MOFs above ×3). Without the two protocols that carry no charges the median is ×3.0 and the 90th percentile ×9.

**Fig. 6:**
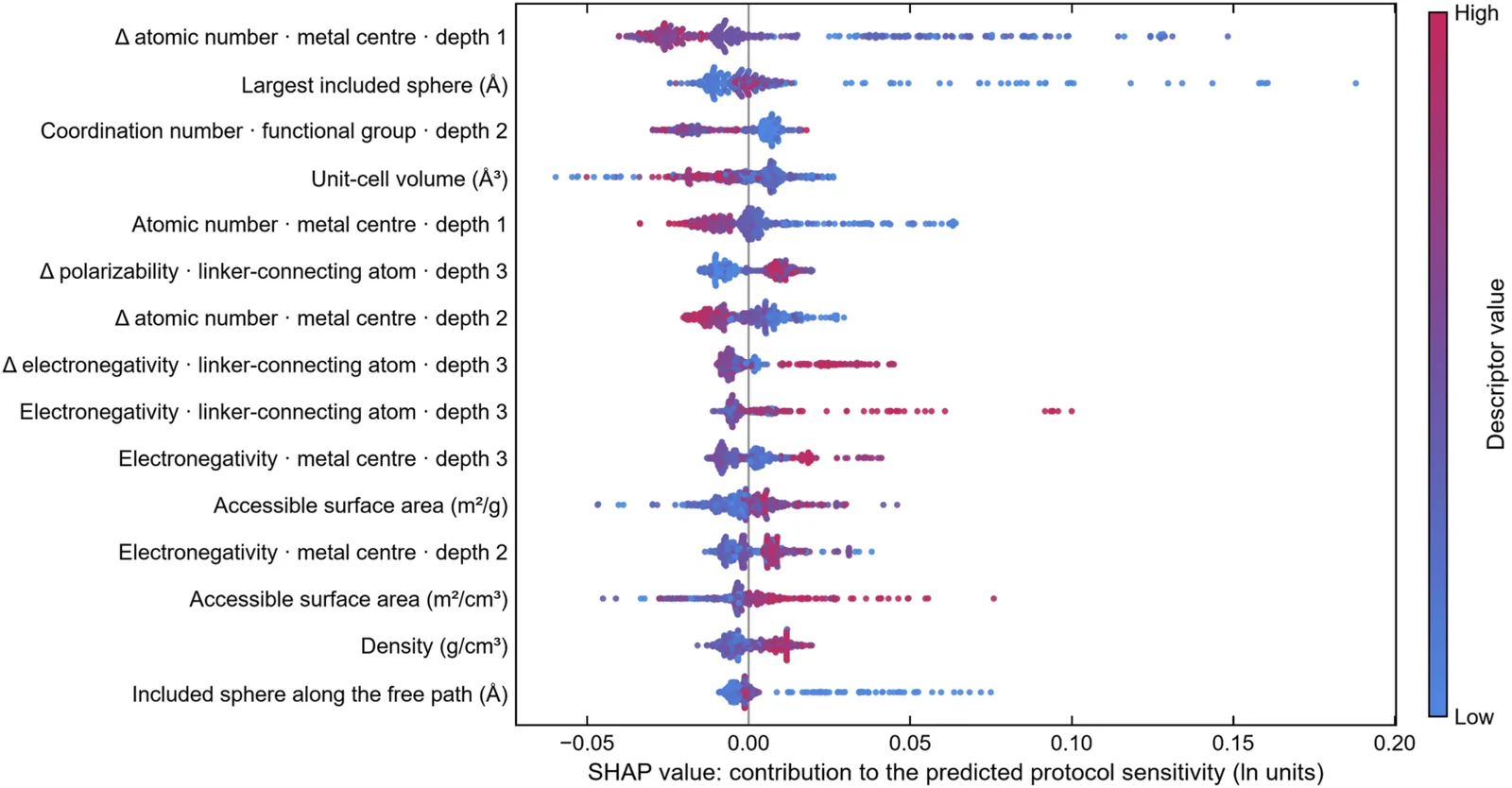
Structural descriptors of protocol sensitivity. SHAP summary of the gradient-boosting model that predicts the protocol sensitivity of a MOF—the standard deviation of its ln(CO_2_ uptake) across the 12 protocols after removal of the protocol main effects, at 298 K and 0.1 bar—from 156 revised-autocorrelation (RAC) and 14 pore descriptors (cross-validated *R*^2^ = 0.43). One dot per MOF; the 15 descriptors with the largest mean |SHAP| are shown in that order, the horizontal position is the contribution to the predicted sensitivity and the colour the descriptor value. RACs are named as property · starting atoms · bond-path depth, Δ marking a difference autocorrelation; the CRAFTED descriptor names are given in Table S23 (SI).

### 3.5 MOFs *versus* COFs

The 667 COFs of CRAFTED contain no metal centres, yet the gauge is not better for them: % GRR at 298 K is 59.4% (95% CI 55.7–63.5%) at 0.1 bar, where only 334 COFs adsorb more than 0.05 mol kg^−1^ under every protocol, 60.0% at 1 bar, 50.9% at 10 bar and 80.4% for selectivity, against 56.1%, 48.3%, 22.2% and 59.4% for the MOFs (Fig. 7). What differs is the source of the disagreement. The protocol main effect—a shift common to all COFs—is larger than for MOFs (sd 0.34 against 0.18 at 1 bar and 0.20 against 0.07 at 10 bar), and per material the UFF–DREIDING gap is 2.0 times the MOF value (median 0.64 against 0.32 ln units) while the charge-scheme spread is half the MOF value (0.20 against 0.39): without metal centres the dispersion parameters, not the charges, drive the disagreement. The material-specific interaction is not smaller, however—at 1 bar its sd is 0.30 for COFs against 0.28 for MOFs—and at 0.1 bar it cannot be separated from the large reported Monte Carlo term (0.51), the artefact resolved for N_2_ in Section 3.8. A calibration of the protocol main effect would therefore remove about 45% of the COF measurement-system variance at 1 bar, but not the rest.

**Fig. 7:**
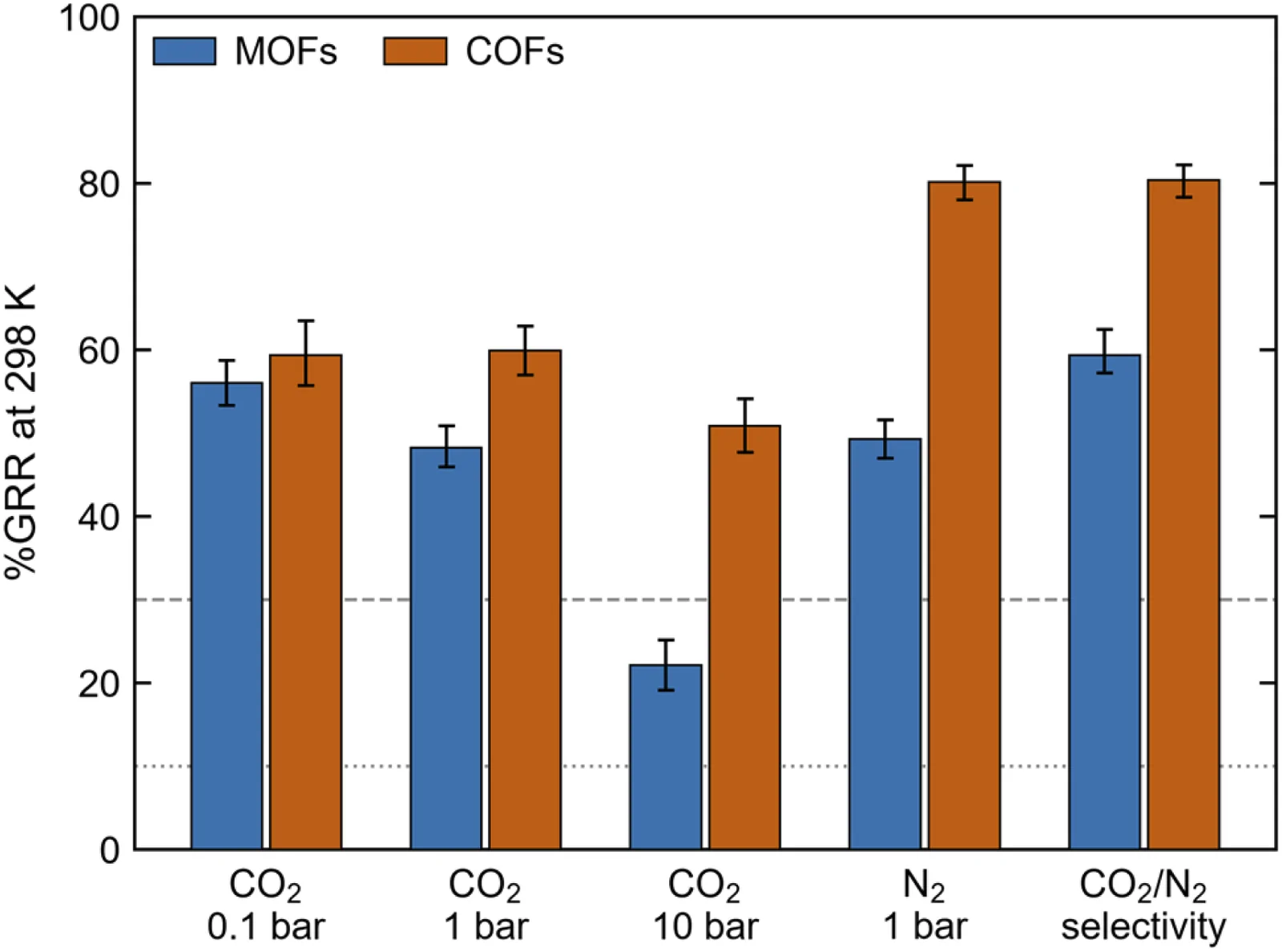
% GRR of the MOFs and the COFs. % GRR at 298 K of the MOFs and of the 667 COFs of CRAFTED for CO_2_ uptake at 0.1, 1 and 10 bar, N_2_ uptake at 1 bar and CO_2_/N_2_ selectivity. Whiskers: bootstrap 95% confidence intervals; dashed and dotted lines: the AIAG thresholds of 30% and 10%. The number of materials at each condition is given in Table 1.

### 3.6 Structural validity

MOFChecker flagged 82 of the 632 MOFs used at 0.1 bar (atomic overlaps, over- or under-coordination, isolated molecules or missing hydrogens). % GRR at 298 K and 0.1 bar is 55.3% (95% CI 52.7–58.4%) on the clean subset and 61.0% (53.2–68.8%) on the flagged subset, against 56.1% for all MOFs (Fig. 8 and Table S25 (SI)). Structural errors do not explain the low resolution, which is a property of the protocol disagreement itself.

**Fig. 8:**
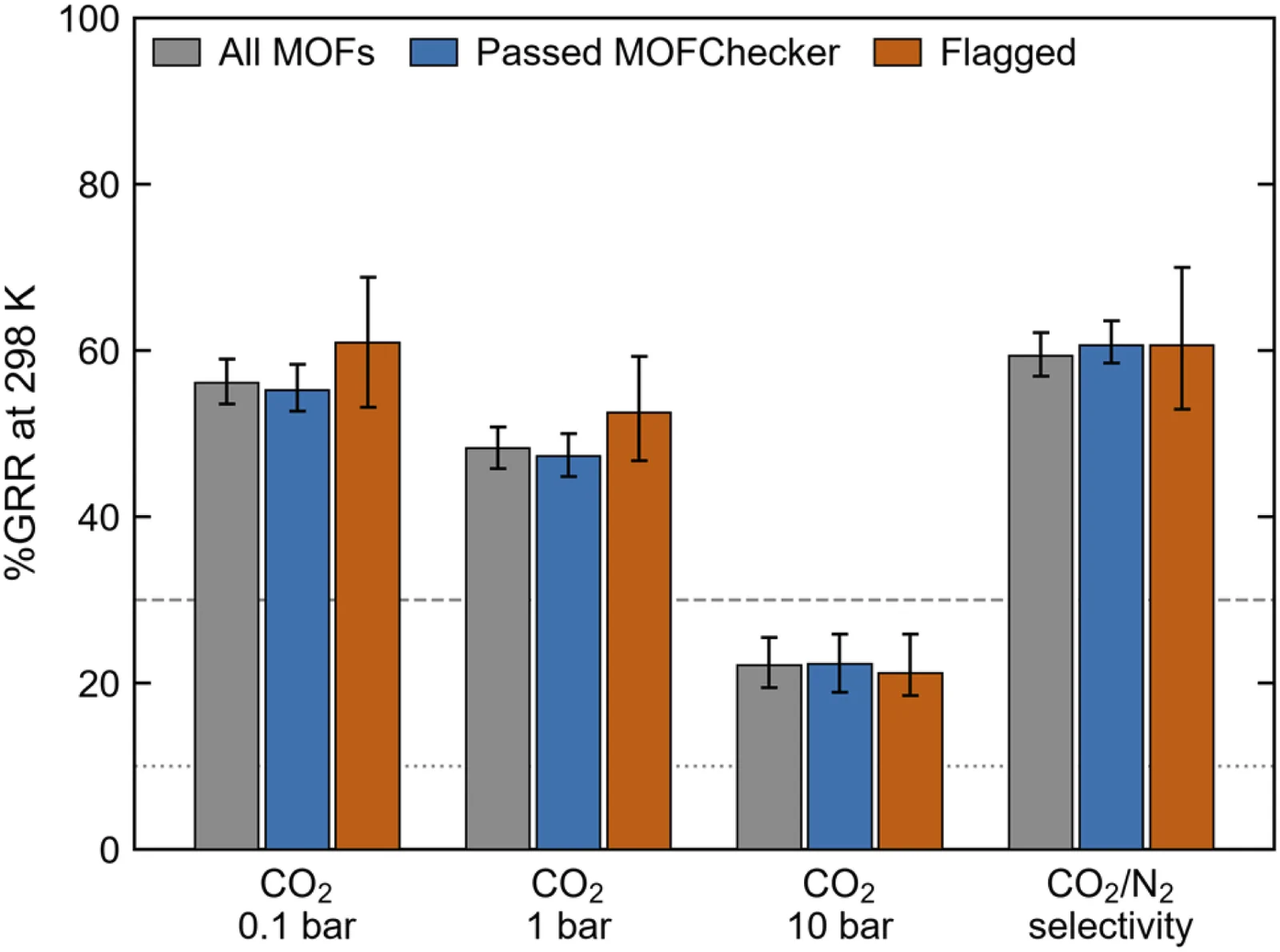
% GRR of the MOFs that pass and that fail MOFChecker. % GRR at 298 K for CO_2_ uptake at 0.1, 1 and 10 bar and for CO_2_/N_2_ selectivity, computed on all MOFs, on the MOFs that pass MOFChecker and on the flagged MOFs (atomic overlaps, over- or under-coordinated atoms, isolated molecules, missing hydrogens). Whiskers: bootstrap 95% confidence intervals (200 resamples); dashed and dotted lines: the AIAG thresholds of 30% and 10%. The subset sizes are given in Table S25 (SI).

### 3.7 Cross-code reproduction

Re-simulating the UFF + DDEC isotherm of MULQOA at 298 K with RASPA3 and the CRAFTED settings reproduced 13 of 13 database points within 2*σ*, with mean *z* = −0.27 and a mean absolute relative difference of 2.2% (Fig. 9 and Table S26 (SI)).

**Fig. 9:**
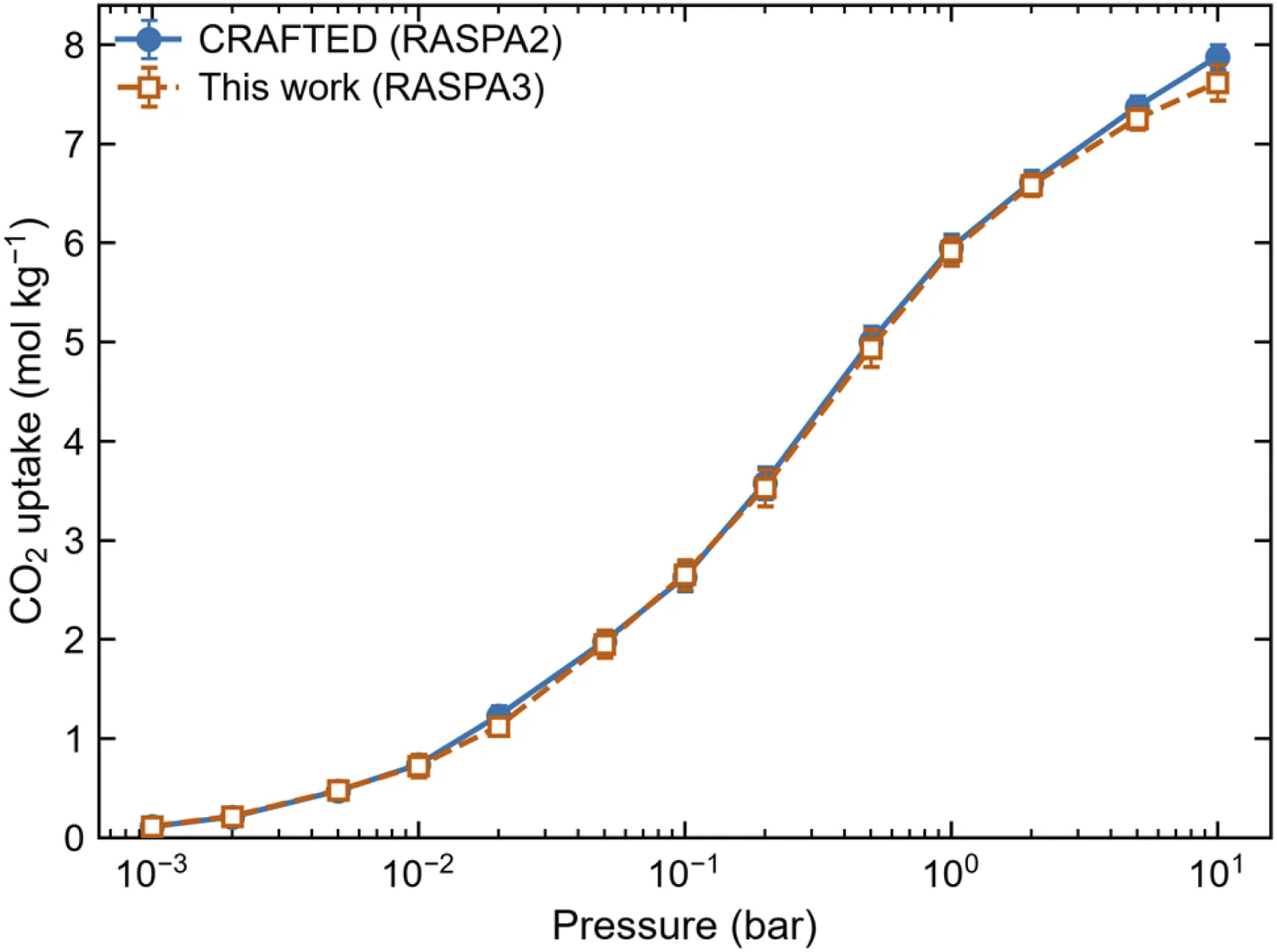
CO_2_ isotherm of MULQOA from CRAFTED and from an independent RASPA3 simulation. UFF + DDEC at 298 K: the database values (RASPA2) and the re-simulation with RASPA3 3.1 and pyMSER truncation, 10 000 cycles per point, each with its Monte Carlo uncertainty.

### 3.8 Factors CRAFTED lacks

#### 3.8.1 Repeatability

Independent pairs of RASPA3 runs of the 30 sampled MOFs give a run-to-run standard deviation of ln(uptake) of 0.041 at 0.1 bar and 0.036 at 1 bar (Fig. 10a and Table S27 (SI)). The MSER uncertainties that the same runs report have medians of 0.086 and 0.038, and CRAFTED reports 0.083 and 0.036 for the same MOFs under the same protocol: like for like, the reported uncertainty overstates the run-to-run scatter by a factor of 2.0 at 0.1 bar and not at all at 1 bar (Table S10 (SI)). The database-wide value used as the repeatability in Section 3.1 (0.157 at 0.1 bar) is larger again, because it pools all 12 protocols, including those with low uptakes and large relative errors; the measured repeatability, in turn, comes from one protocol and stands for all twelve. Quadrupling the sampling of N_2_ at 1 bar leaves the reported uncertainty unchanged (ratio 1.01, where a standard error would fall to 0.50) and moves the mean by +0.009 ± 0.028 ln units: for N_2_ the reported ‘uncorrelated standard deviation’ is the fluctuation amplitude of the equilibrated trace, not the uncertainty of its mean, and it is nine times the run-to-run scatter of the same MOFs.

**Fig. 10:**
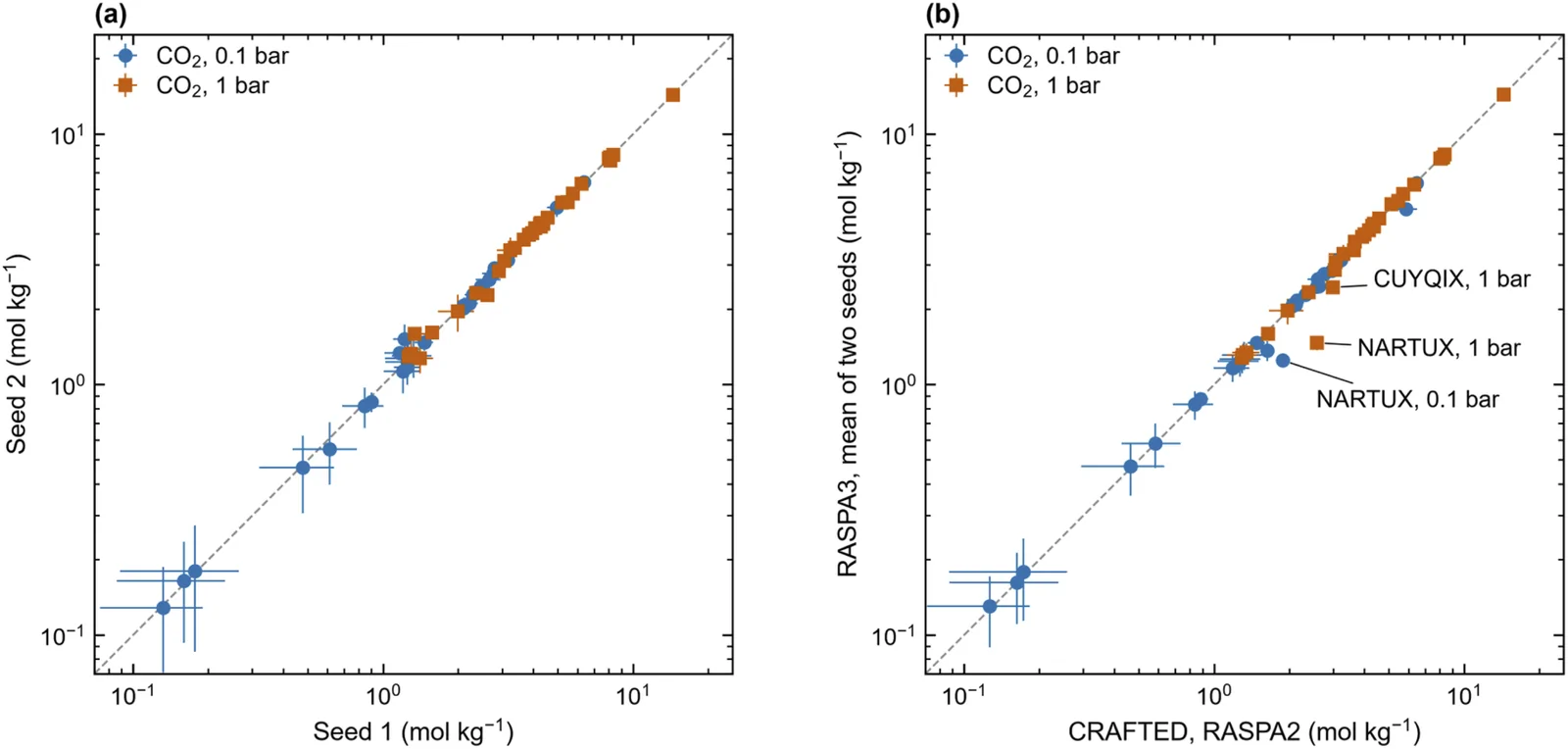
Repeatability and code effect for the 30 sampled MOFs. CO_2_ uptake at 298 K and 0.1 or 1 bar (UFF + DDEC). (a) Two RASPA3 runs with different random seeds; error bars: the MSER uncertainty reported by each run, which at 0.1 bar is about twice the run-to-run scatter (Section 3.8). (b) Mean of the two seeds against the CRAFTED (RASPA2) value; the labelled conditions differ from the database by more than 2*σ* and are examined in Fig. 11. Dashed lines: equality.

#### 3.8.2 Code

The mean of the two RASPA3 seeds differs from the database by −0.031 ± 0.084 ln units at 0.1 bar and −0.031 ± 0.108 at 1 bar (mean ± sd across MOFs), and 29 and 28 of 30 MOFs agree within 2*σ* (Fig. 10b). The three exceptions—NARTUX at 0.1 bar, CUYQIX at 1 bar and NARTUX at 1 bar, all in the most protocol-sensitive group—were re-run for 40 000 cycles with a third seed: their uptake rises by +0.30 ln units on average and all end within 2*σ* of the database (mean ln difference −0.39 → −0.09), whereas four well-behaved controls move by +0.008 (Fig. 11 and Table S28 (SI)). The apparent code effect is slow equilibration of strongly bound CO_2_ in narrow, highly charged pores, which the MSER criterion does not detect within 10 000 cycles.

**Fig. 11:**
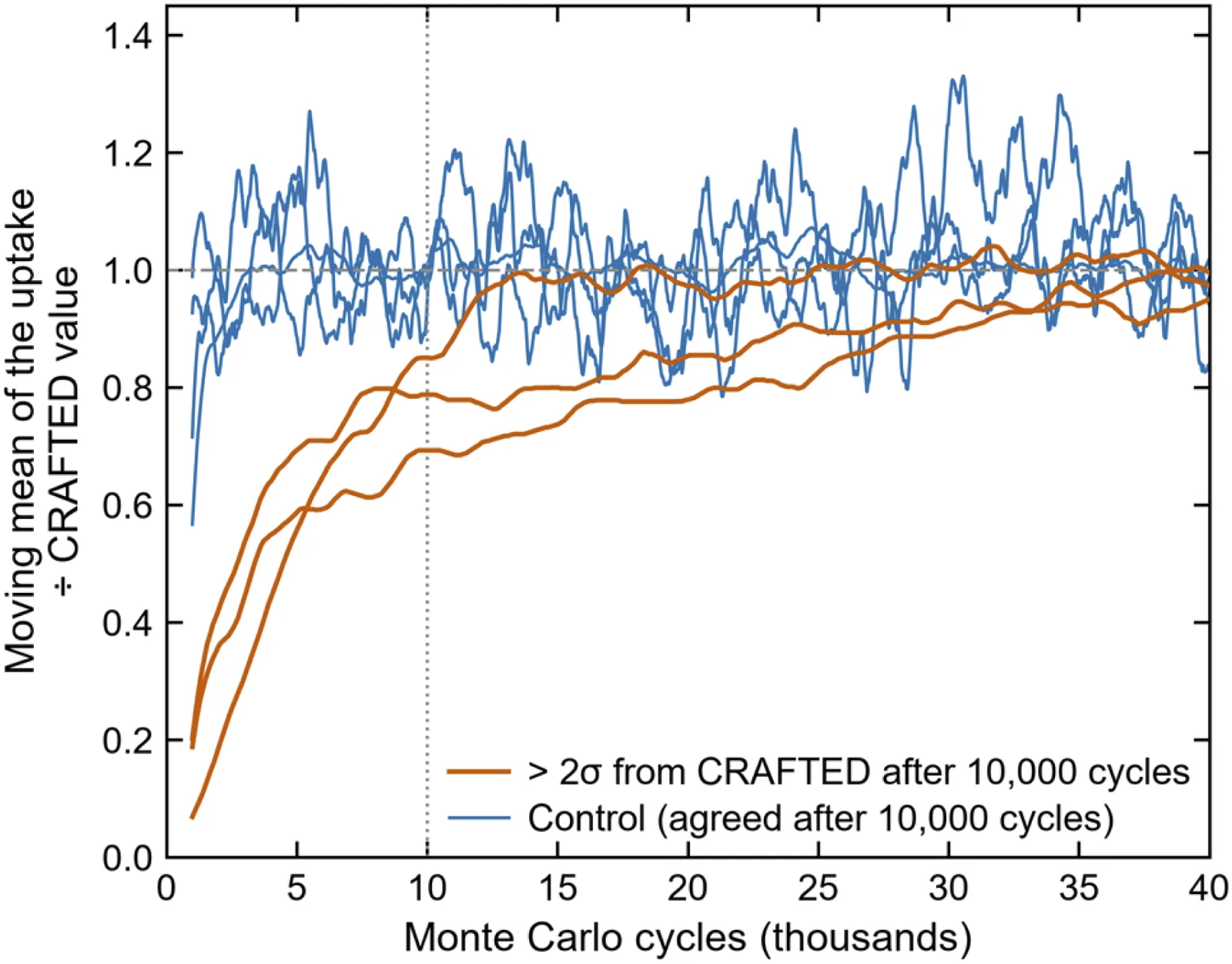
Long runs of the conditions that disagreed with the database. 1000-cycle moving mean of the CO_2_ uptake divided by the CRAFTED value in 40 000-cycle RASPA3 runs (third seed) of the three conditions that differed from the database by more than 2*σ* after 10 000 cycles (NARTUX at 0.1 bar, CUYQIX at 1 bar and NARTUX at 1 bar) and of four controls that agreed. Dotted line: the CRAFTED run length; dashed line: the CRAFTED value.

#### 3.8.3 Sorbate model and cut-off

Replacing TraPPE CO_2_ by EPM2 changes the uptake at 0.1 bar by −0.056 ± 0.084 ln units (×0.95; 29 of 30 MOFs inside the Monte Carlo noise). Replacing CRAFTED's 12.8 Å shifted potential by a 12.0 Å truncated potential with tail corrections changes it by +0.228 ± 0.156 ln units (×1.26; only 17 of 30 inside the noise)—a mean shift comparable with the DREIDING-versus-UFF main effect of the database (−0.240), from a setting that screening papers often leave unreported (Fig. 12, Tables 3 and S29 (SI)). The 30 MOFs over-represent the extremes of protocol sensitivity, and the shift is largest in the most sensitive group (+0.287, against +0.197 and +0.200 in the least sensitive and middle groups; Table S9 (SI)), so +0.228 is a mean over the sample rather than over the database; a typical MOF, from the middle group, moves by +0.200 (×1.22).

**Fig. 12:**
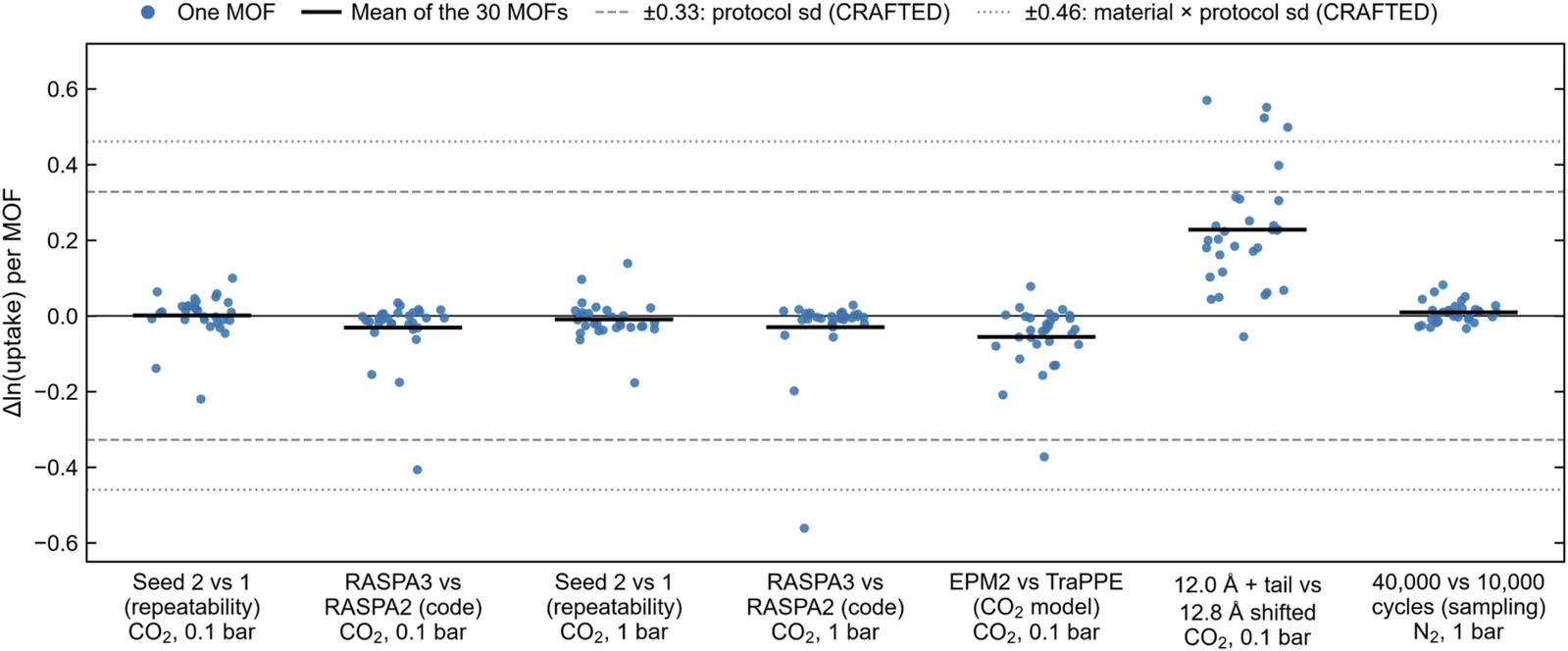
Effect of the factors that CRAFTED holds fixed, measured on 30 MOFs. Change of ln(uptake) of each MOF (UFF + DDEC, 298 K) when one factor is varied in RASPA3: a second random seed (repeatability), RASPA3 instead of CRAFTED's RASPA2 (code), EPM2 instead of TraPPE CO_2_ (CO_2_ model), a 12.0 Å truncated cut-off with tail corrections instead of the 12.8 Å shifted potential (cut-off; separated into its distance and treatment parts, which do not add, in Tables S30 and S31 (SI)) and 40 000 instead of 10 000 cycles (sampling; N_2_ at 1 bar). The 30 MOFs are the 10 most and the 10 least protocol-sensitive and 10 drawn at random from the middle quartiles, so the means are those of the sample, not of the database (Table S9 (SI)). Black bars: mean over the MOFs; dashed and dotted lines: CRAFTED's protocol sd (±0.33) and interaction sd (±0.46) for CO_2_ at 0.1 bar. The values are listed in Table 3.

**Fig. 13:**
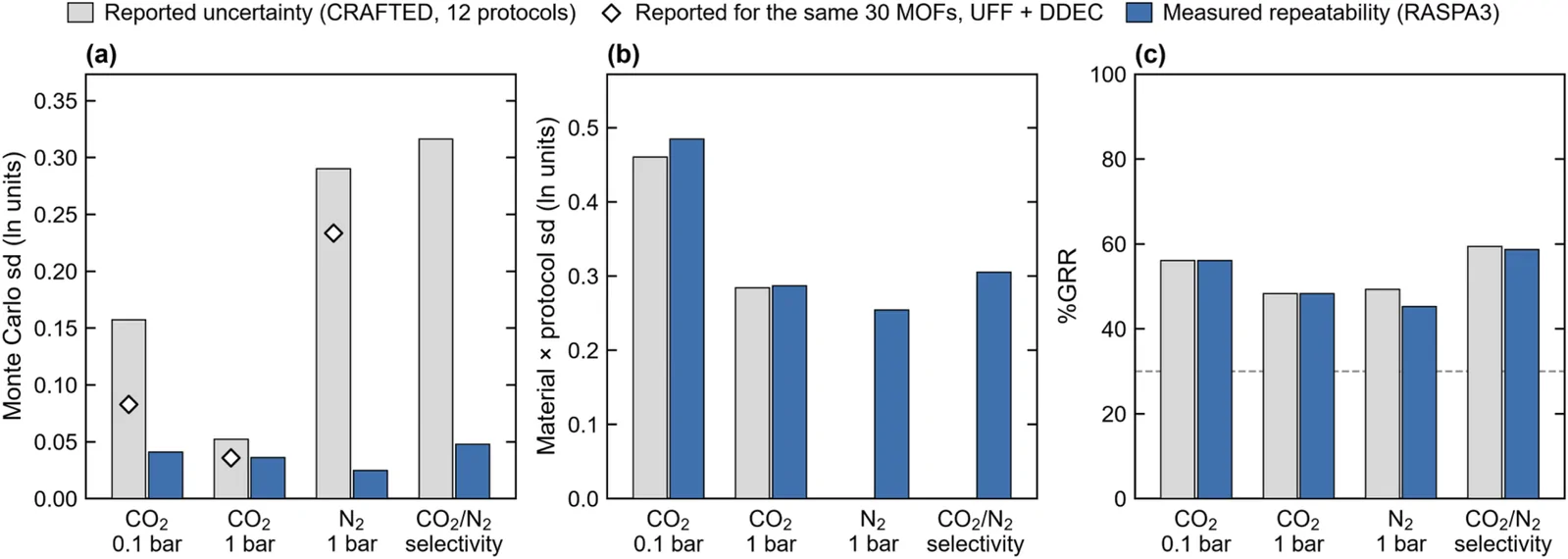
Variance decomposition with the measured repeatability. The decomposition of Fig. 2 (MOFs, 298 K) with the run-to-run repeatability measured with the RASPA3 replicates in place of CRAFTED's reported uncertainty, for CO_2_ at 0.1 and 1 bar, N_2_ at 1 bar and CO_2_/N_2_ selectivity: (a) Monte Carlo sd, (b) material × protocol interaction sd and (c) % GRR, with the AIAG 30% threshold dashed. Grey bars: the reported uncertainty pooled over the 12 protocols, as used in Fig. 2 and Table 1; diamonds in (a): the uncertainty CRAFTED reports for the same 30 MOFs under UFF + DDEC, the like-for-like counterpart of the measured value (Table S10 (SI)); a missing grey bar in (b) is an interaction clipped at zero. NDC is the same with either repeatability (Table 4).

**Table 3:** Factors that CRAFTED holds fixed, measured with RASPA3 on 30 MOFs (UFF + DDEC, 298 K): mean and across-MOF sd of the change in ln(uptake), run-to-run sd from replicate seeds and number of MOFs whose change lies inside the combined Monte Carlo noise (2*σ*). The last three rows give the CRAFTED variance components at the same condition for comparison. The 30 MOFs over-represent the extremes of protocol sensitivity; the means by group are in Table S9 (SI). The complete table is Table S29 (SI); per-MOF values are in the SI CSV files

Factor varied	Condition	*n*	Mean Δ ln *u*	sd across MOFs	Run-to-run sd	Inside 2*σ*
Replicate seed (seed 2 *vs.* 1)	CO_2_, 0.1 bar	30	+0.001	0.059	0.041	
Replicate seed (seed 2 *vs.* 1)	CO_2_, 1 bar	30	−0.010	0.051	0.036	
Code (RASPA3 *vs.* RASPA2)	CO_2_, 0.1 bar	30	−0.031	0.084		29
Code (RASPA3 *vs.* RASPA2)	CO_2_, 1 bar	30	−0.031	0.108		28
Sampling (40 000 *vs.* 10 000 cycles)	N_2_, 1 bar	30	+0.009	0.028		30
CO_2_ model (EPM2 *vs.* TraPPE)	CO_2_, 0.1 bar	30	−0.056	0.084		29
Cut-off (12.0 Å + tail *vs.* 12.8 Å shifted)	CO_2_, 0.1 bar	30	+0.228	0.156		17
Long run, discrepant conditions (40 000 *vs.* 10 000)	CO_2_, 0.1/1 bar	3	+0.298	0.120		3
Long run, control conditions (40 000 *vs.* 10 000)	CO_2_, 0.1/1 bar	4	+0.008	0.016		4
CRAFTED: protocol main effect	CO_2_, 0.1 bar	632		0.328		
CRAFTED: material × protocol interaction	CO_2_, 0.1 bar	632		0.460		
CRAFTED: Monte Carlo (reported)	CO_2_, 0.1 bar	632		0.157		

#### 3.8.4 Which part of the cut-off convention

That comparison moves two settings at once, so the 2 × 2 design of Section 2.8 separates them on the same 30 MOFs (Table S30 (SI)). The two settings do not act independently. With tail corrections the distance has no effect: 12.0 Å and 12.8 Å give the same uptake (+0.002 ± 0.048 ln units). With the shifted potential it does: shortening the cut-off to 12.0 Å changes ln(uptake) by −0.062 ± 0.046, and lengthening it to 14.0 Å by +0.050. The treatment effect therefore depends on the distance—+0.226 ± 0.139 at CRAFTED's 12.8 Å and +0.289 at 12.0 Å (Table S31 (SI))—and no share of the combined shift belongs to either setting alone. This is the pattern the potentials lead one to expect: the shifted potential discards the attraction beyond the cut-off and raises the well inside it, a loss that shrinks as the cut-off grows, while the tail correction restores the missing attraction, so that the result no longer depends on the distance, as Jablonka *et al.* showed for porous materials.^34^ CRAFTED's convention thus gives uptakes at 0.1 bar 20% below the tail-corrected value (×0.80), more in the most sensitive group (×0.74) than in the least sensitive one (×0.83). What a screening paper must report is therefore the treatment of the potential beyond the cut-off and, with a shifted potential, the distance as well—the part most often left unsaid.

#### 3.8.5 Measured repeatability

With the measured repeatability in place of the reported uncertainty (Table 4 and Fig. 13), % GRR for CO_2_ at 0.1 bar is unchanged (56.1%) and the interaction sd rises from 0.46 to 0.48; for N_2_ at 1 bar the interaction, previously clipped at zero, becomes 0.25 and % GRR moves from 49.3% to 45.2%; for selectivity the interaction becomes 0.31 and % GRR 58.7%. NDC does not change. The low resolution for N_2_ and selectivity is therefore protocol disagreement as well, and more sampling would not repair it.

**Table 4:** The variance decomposition of Table 1 (MOFs, 298 K) repeated with the measured run-to-run repeatability (RASPA3 replicate seeds; for N_2_ from the 10 000- *versus* 40 000-cycle difference; for selectivity combined in quadrature) in place of CRAFTED's reported Monte Carlo uncertainty. Standard deviations in ln units

Response	*P*/bar	*n*	sd MC reported	sd MC measured	sd interaction reported	sd interaction measured	% GRR reported	% GRR measured	NDC reported	NDC measured
CO_2_	0.1	632	0.157	0.041	0.460	0.485	56.1	56.1	2	2
CO_2_	1	669	0.053	0.036	0.284	0.287	48.3	48.3	2	2
N_2_	1	614	0.290	0.025	0.000	0.254	49.3	45.2	2	2
CO_2_/N_2_ selectivity		613	0.316	0.048	0.000	0.305	59.4	58.7	1	1

### 3.9 Experimental anchor

Of the 690 CoRE MOF 2014 structures in CRAFTED, seven have public experimental pure-CO_2_ isotherms at 296–300 K in the NIST/ARPA-E database: IRMOF-1 (MOF-5), ZIF-8, ZIF-90, Zn-MOF-74, MIL-53(Al), NOTT-300 and DMOF-1 [Zn_2_(bdc)_2_(dabco)]. The most-measured MOFs—HKUST-1, UiO-66 and Mg-MOF-74—are absent, because the database contains no Cu-carboxylate, Zr or Mg frameworks. Nine published isotherms give twelve experimental values at 0.1 and 1 bar (Fig. 14 and Table S32 (SI)). The measurement lies inside the range spanned by the 12 protocols for one of five values at 0.1 bar and five of seven at 1 bar; the median |ln(measured/reference protocol)| is 0.78 at 0.1 bar and 0.52 at 1 bar, against median protocol ranges ln(max/min) of 1.69 and 0.81. The largest deviations have opposite signs: the reference protocol over-predicts NOTT-300 by a factor of 5.9 at 0.1 bar, under-predicts Zn-MOF-74 by a factor of 3.9 at 0.1 bar and over-predicts DMOF-1 by a factor of 2.4 at 1 bar. The under-prediction for Zn-MOF-74 is the known failure of generic force fields at open metal sites, where CO_2_ binds more strongly than Lennard-Jones and point-charge terms describe.^35^ Agreement between protocols (precision) is therefore not a bound on the disagreement with experiment (accuracy), and the sign of the error is material-specific.

**Fig. 14:**
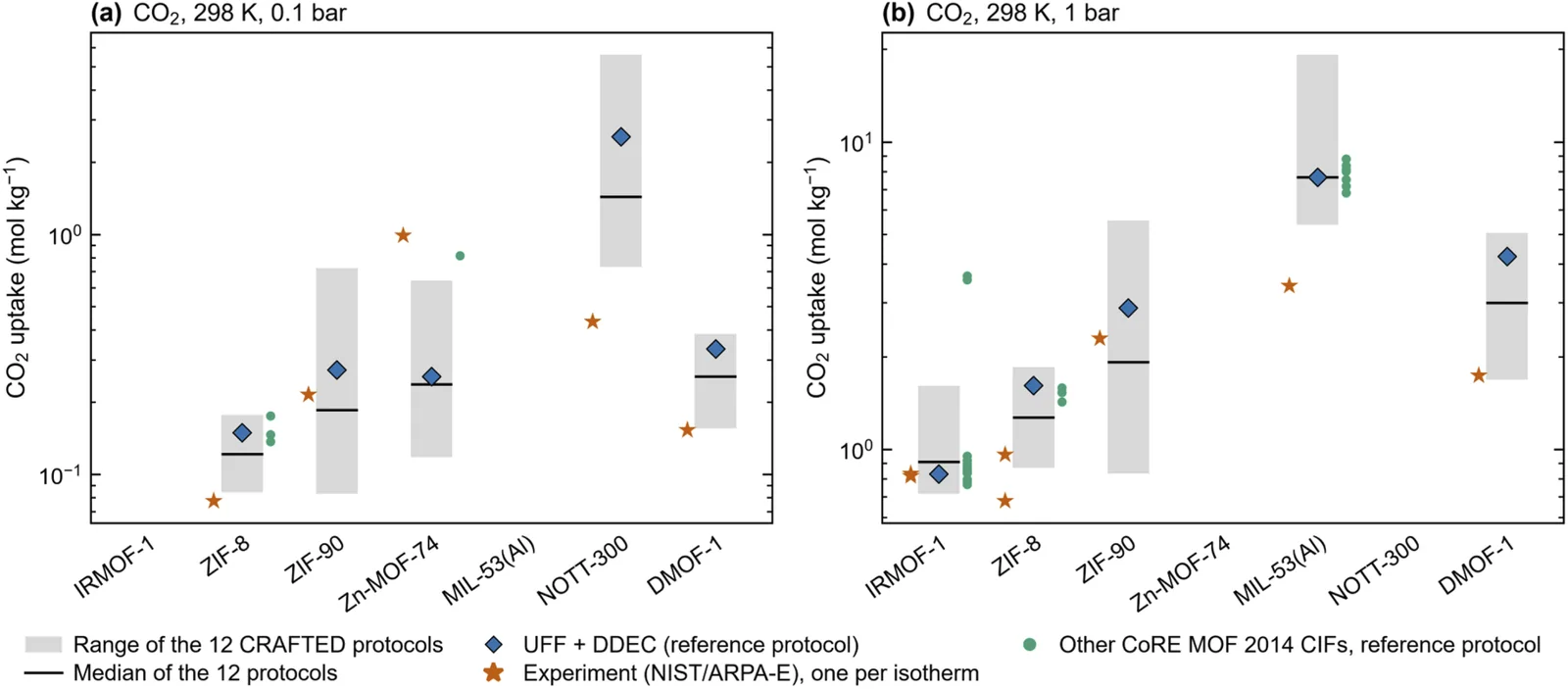
Experimental CO_2_ uptake against the range of the 12 protocols. The seven CRAFTED MOFs with public experimental pure-CO_2_ isotherms at 296–300 K in the NIST/ARPA-E database, at 298 K and (a) 0.1 bar, (b) 1 bar. Grey bars: range of the 12 CRAFTED protocols, with their median; diamonds: the reference protocol (UFF + DDEC); stars: published isotherms interpolated at the pressure, one per isotherm; green dots: other CoRE MOF 2014 CIFs of the same composition under the reference protocol. An empty slot means that no published isotherm brackets that pressure; the comparison widened to 273–323 K and 10 bar is in Table S37 (SI). MIL-53(Al) breathes, which the rigid CRAFTED structure cannot represent, and its protocol range reaches 19.2 mol kg^−1^ under the Qeq protocols (Section 3.4).

Isotherms from different laboratories scatter as well:^36^ where two groups measured the same material, the between-laboratory sd is 0.01 ln units for IRMOF-1 (MOF-5) at 1 bar (between protocols: 0.24) and 0.25 ln units for ZIF-8 at 1 bar (between protocols: 0.31) (Table S33 (SI)). A further hidden factor appears in the same comparison: CoRE MOF 2014 holds several CIFs of the same composition, and under the reference protocol the 22 CIFs of IRMOF-1 (MOF-5) span ln(max/min) = 1.56 and the two CIFs of Zn-MOF-74 span ln(max/min) = 1.16 (against 0.25–0.26 for MIL-53(Al) and ZIF-8)—a structure-source effect as large as the protocol effect (Table S34 (SI)).

Seven materials are few, so two questions follow: whether they are representative, and whether more public data change the picture. On protocol sensitivity they span the database rather than avoiding its difficult end—their percentiles run from 8 to 97 with a median of 57, and three of seven fall in the most protocol-sensitive third (Table S35 (SI)). Within that third the gauge is worse, not better: % GRR at 0.1 bar is 71.7% with NDC 1, against 40.8% and NDC 3 in the least sensitive third (Table S36 (SI)), so a benchmark weighted towards protocol-sensitive materials would widen the gap between simulation and measurement rather than close it. Taking every public isotherm of these materials at the database's three temperatures and at 0.1, 1 and 10 bar raises the comparison from twelve values to 31 (18 isotherms from 13 papers; Table S37 (SI); the isotherms and their sources are listed in Table S38 (SI)). The measurement lies outside the whole 12-protocol range in 20 of them, but these values are neither independent nor all far outside (Table S11 (SI)). Ten are MIL-53(Al), the one framework in the set that breathes, which the rigid structures of the database cannot represent at all, and eight of its ten values come from three isotherms of one paper at 323 K. For the rigid frameworks the measurement lies outside the range in 10 of 21 cases, only two of them by more than 0.25 ln units, and nine of the ten lie below it: the measurement is lower than every protocol. A gap of one sign is what samples that are less than ideal crystals produce—incomplete activation, defects, residual solvent—as much as what a force field produces, and the anchor cannot tell the two apart. For the same reason it cannot rank the protocols: the reference protocol is the farthest from the measurements (median |ln(measured/simulated)| 0.52 for the rigid frameworks, against 0.34 for the median of the 12 protocols), because the no-charge protocols pull the median down towards the low measurements. Where two or more groups measured the same material at the same condition, the standard deviation between laboratories has a median of 0.18 ln units, against 0.28 between protocols at the same four conditions (ranges ln(max/min) 0.29 and 0.78; Table S39 (SI)): the experimental side carries real uncertainty too, and at IRMOF-1 (MOF-5), 298 K and 10 bar it is the larger of the two.

## 4 Discussion

The measurement-systems framing turns a familiar qualitative statement—‘force fields and charges matter’—into a quantitative resolution claim with a confidence interval. Judged by the AIAG criteria,^6^ GCMC screening of CO_2_ uptake is a measurement system that is unacceptable at 0.1 and 1 bar and marginal at 10 bar (% GRR 56%, 48% and 22% at 0.1, 1 and 10 bar); for CO_2_/N_2_ selectivity it cannot separate materials into more than one class at any temperature. The bootstrap intervals are about ±3 percentage points wide when the materials are resampled and about ±8 when the protocols are resampled as well, and every verdict holds under both, so none depends on which MOFs, or which of the protocols in use, happen to be in the database.

Two features of the decomposition matter for practice. First, the interaction dominates at low pressure, so no protocol-level calibration—a scale factor or a linear correction—can restore resolution; the error is material-specific and must be modelled as such, which is the subject of a follow-up study on bias against experiment. Second, the Monte Carlo uncertainty reported by the database is not the run-to-run scatter: for N_2_ it is the fluctuation amplitude of the trace rather than a standard error—four times longer runs leave it unchanged, and it is about nine times the scatter of independent runs—and for CO_2_ it is twice that scatter at 0.1 bar and about right at 1 bar. With the measured repeatability the N_2_ and selectivity errors are also material-specific protocol disagreement, so more computing does not buy resolution anywhere in the low-pressure regime. Screening studies that carry reported Monte Carlo uncertainties into rankings should measure the repeatability from replicate runs instead.

The decision analysis shows what the variance numbers mean for a screening paper: of a top-10 list produced at 0.1 bar with one protocol, on average only three members remain in the top-10 of another reasonable protocol, and no MOF is top-10 under every protocol. Rankings from single-protocol screens at low pressure should therefore be read as protocol-conditional. The sensitivity model offers a constructive way forward: almost half of the per-material sensitivity is predictable from structure, so a screen can flag in advance the candidates whose ranking is least trustworthy and direct higher-fidelity effort—DFT-derived charges, polarizable or *ab initio* force fields, or experiment—to them.

The COF comparison refines the mechanism. Removing the metal centres does not make the gauge acceptable; it moves the dominant source of disagreement from the charge scheme to the dispersion parameters and turns a larger share of the error into a global protocol shift that a calibration could remove. The material-specific part, however, remains as large as for MOFs, so the resolution problem is not confined to metal sites.

Of the factors that screening databases hold fixed, the simulation code, the random seed and the CO_2_ model are small next to the protocol effects, but the cut-off convention is not: CRAFTED's shifted potential gives uptakes about 20% below tail-corrected ones at 0.1 bar, and by different amounts for different MOFs. The treatment of the potential beyond the cut-off is what matters, as Jablonka *et al.* found:^34^ with tail corrections the distance hardly matters, with a shifted potential it does, so reporting the distance alone—the usual practice where anything is reported at all—does not pin the number down. Both belong in the methods section of every screening study.

### 4.1 Scope, and what would change the answer

The appraisers here are the 12 generic combinations that high-throughput screens actually use; the conclusions are about that family, on rigid frameworks, for pure CO_2_ and N_2_. Narrowing the family helps but does not rescue it: with only the DFT-derived and machine-learned charge schemes % GRR is 33% rather than 56% and NDC 4 rather than 2, and that is better agreement among three schemes that share one target, not a demonstrated gain in accuracy (Section 3.1). Material-specific or *ab initio*-derived force fields^35^ address exactly the interaction term measured here, and for the open-metal-site frameworks where generic parameters fail they are the right answer; but they are not available for a thousand materials at once, and the choices inside them—reference method, basis set, fitting set—would become appraisers of their own. The measurement-systems question would then have to be asked again of that family. Flexibility, mixtures and water, all absent from CRAFTED, can only add variance, not remove it.

### 4.2 Practical guidance under a compute budget

A screen that cannot afford twelve protocols can still avoid the worst of this; each recommendation below was tested on the CRAFTED data (Table S40 (SI)). (a) Use charges derived from or trained on DFT: at no extra GCMC cost the protocols agree far better (% GRR 33% rather than 56%), but they are not shown to be closer to experiment, and their narrower range is overconfident: it contains the measurement for four of the 21 values of rigid frameworks, against 11 for all 12 protocols. (b) Run two protocols rather than one and report the per-material interval with the ranking, but do not read it as the protocol uncertainty: two protocols chosen at random span a median of 32% of a MOF's 12-protocol range, and even the pair with the most different mean biases (UFF + Qeq and DREIDING + no charges) spans 83% of it and contains the whole range for only 28% of the MOFs; because of the interaction, no fixed pair brackets every material. (c) Before committing to a full screen, run the same protocols on a pilot of 20–30 materials: a random pilot of 30 MOFs with all 12 protocols reproduces the verdict of the whole database in every one of 1000 draws (% GRR 45–69% at 0.1 bar) and says whether the candidates can be separated at all. (d) Short-list on rank robustness—the materials that stay in the top-*k* under both protocols—and expect a short list: two protocols' top-10 lists share a median of 2.5 MOFs, and none for 24 of the 66 pairs, but a shared member is in a third protocol's top-10 44% of the time, against 25% for a member of one protocol's list. (e) Use the structure-based sensitivity model to decide where the second protocol or higher-fidelity treatment is worth spending: cross-validated, 71% of the MOFs it places in the most sensitive third belong there (a third would by chance), and 39% of those it places in the most sensitive tenth. (f) Report the cut-off distance and its treatment, the sampling length and the repeatability measured from replicate runs, and screen the uptakes for physical plausibility (Section 2.1).

### 4.3 Limitations

CRAFTED uses rigid frameworks and one CIF per database entry. The hidden factors were quantified on 30 MOFs chosen to span the sensitivity ranking—the cut-off convention in a full 2 × 2 design, the others one at a time—and the structure source (CoRE MOF 2014 *versus* CSD-derived CIFs) and framework flexibility remain to be tested systematically. Gauge R&R quantifies precision, not accuracy: the experimental anchor rests on the seven CRAFTED materials with public isotherms, and for the rigid frameworks the measurement lies outside the whole protocol range in 10 of 21 comparisons, mostly just below it. That anchor is drawn from heterogeneous literature isotherms, not from one controlled campaign; the measurements that would settle the accuracy question are a consistent set on materials chosen by protocol sensitivity rather than by fame, which this paper can now specify but not perform, and a bias model calibrated against curated experimental data is the next step of this work.

## 5 Conclusions

Treating one widely used family of GCMC screens as a measurement system shows that, at flue-gas conditions, the measurement system accounts for 32% of the variance in predicted CO_2_ uptake among 632 rigid MOFs (56% of the study variation) and resolves only two distinct classes of material, and no more than four when the family is narrowed to DFT-derived and machine-learned charges; these verdicts hold when the protocols, as well as the materials, are treated as a sample. The error is dominated by a material-specific interaction rather than a global bias, so that top-*k* short-lists are largely artefacts of the protocol at 0.1 bar and protocol-conditional at 1 bar. Protocol sensitivity is partly predictable from pore size, metal environment and linker chemistry, and metal-free COFs are measured no better. Replicate runs scatter half as much as the reported uncertainty for CO_2_ at 0.1 bar and about a ninth as much for N_2_, so more sampling cannot repair the resolution; CRAFTED's shifted-potential cut-off gives uptakes about 20% below tail-corrected ones; and for 10 of 21 published values of rigid frameworks the measurement lies outside the whole protocol range, mostly just below it. Screening studies should report % GRR and NDC—or at minimum the top-*k* overlap between two protocols—alongside any ranking, state both the cut-off distance and its treatment, screen their uptakes for physical plausibility, and treat agreement between protocols as precision, not accuracy.

## Author contributions

M. M. Hossain: conceptualization, methodology, software, validation, formal analysis, investigation, data curation, visualization, writing – original draft, writing – review & editing.

## Conflicts of interest

There are no conflicts to declare.

## Supplementary Material

RA-OLF-D6RA09230F-s001

## Data Availability

Data analysed in this work are openly available from their original repositories. The CRAFTED database, version 2.0.1 (simulated CO_2_ and N_2_ isotherms of 690 CoRE MOF 2014 and 667 CURATED COF structures under the 12 force-field and charge-scheme protocols, with the crystal structures and descriptors used here), is available on Zenodo at https://doi.org/10.5281/zenodo.10120180 under the CDLA-Sharing-1.0 licence.^8^ The 18 experimental CO_2_ isotherms used in Section 3.9 (13 publications) are available from the NIST/ARPA-E Database of Novel and Emerging Adsorbent Materials (NIST Standard Reference Database 205) at https://doi.org/10.18434/T43882;^13^ their database identifiers and the DOIs of the original publications are listed in Table S38 (SI). Data generated in this work—the results of the 330 new RASPA3 simulations of 30 MOFs (the 240 runs of the one-factor-at-a-time design and the 90 runs that complete the 2 × 2 cut-off design) and of the seven 40 000-cycle re-runs (one record per run with its settings, uptake and uncertainty, and the per-cycle loading traces of the long runs), the 13-point cross-code reproduction of the MULQOA isotherm, the MOFChecker flags of the 690 MOF structures, every result table behind the figures and tables, and CSV versions of all SI tables—are available on Zenodo at https://doi.org/10.5281/zenodo.23990965 under the CC BY 4.0 licence (values copied from CRAFTED keep its CDLA-Sharing-1.0 licence).^33^ The SI tables are also supplied as CSV files with this article. The code for the complete analysis—the RASPA3 simulation worker, the scripts for every analysis step, the figure module and the manuscript generator—can be found at https://doi.org/10.5281/zenodo.23990965 under the MIT licence.^33^ The version of the code employed for this study is version 1.0.0. The simulations used RASPA3 3.1 (ref. 12) and pyMSER,^23^ and the structures were checked with mofchecker-next 0.1.2;^31^ all three are openly available. The version of every Python package is listed in Table S12 (SI) and in the deposit's requirements file, and the deposit's README gives one command per step (the new simulations take about 160 CPU-hours; every other step runs in minutes on a laptop). No data or code are withheld. Supplementary information (SI): steps to reproduce every result, settings of the new RASPA3 simulations, Tables S1–S40 and machine-readable CSV versions of all tables. See DOI: https://doi.org/10.1039/d6ra09230f.
