# Supplementary material for: Quantifying force-field and partial-charge uncertainty in high-throughput grand canonical Monte Carlo screening of metal–organic frameworks for CO2 capture
Source: RSC Adv. 2026 Nov 13. Online ahead of print. doi: 10.1039/d6ra09230f (PMC13765527; doi:10.1039/d6ra09230f)
Supplement: Supplementary file 1 [file RA-OLF-D6RA09230F-s001.pdf]

Supplementary Information (SI) for RSC Advances.
This journal is © The Royal Society of Chemistry

Electronic Supplementary Information

Quantifying force-field and partial-charge uncertainty in high-throughput grand canonical Monte Carlo screening of metal–organic frameworks for CO_2 capture†

Mohammad Masrur Hossain, Department of Industrial and Systems Engineering, The University of Tennessee, Knoxville, TN 37996, USA. E-mail: masrurhossain99@gmail.com

Contents

S1 Reproducing every result in the article

S2 Settings of the new RASPA3 simulations

Table S1 Variance components for CO_2 uptake of MOFs at 298 K and 0.1 bar by the method of moments and by REML (statsmodels MixedLM).

Table S2 Software environment of the analysis (captured when the manuscript was built).

Table S3 Variance components (sd, ln units), %GRR and NDC with bootstrap 95% confidence intervals for every class, temperature, response and pressure.

Table S4 Protocol main effects at 298 K: mean change of ln(uptake) relative to UFF + DDEC, the corresponding factor on the uptake and its sd across materials.

Table S5 Agreement of the top-k short-lists of the 12 protocols (MOFs) for every temperature, response, pressure and k.

Table S6 Per-MOF protocol sensitivity (CO_2, 298 K, 0.1 bar) of the 20 most and the 20 least sensitive MOFs; all MOFs are in the CSV file.

Table S7 Five-fold cross-validated performance of the gradient-boosting models of protocol sensitivity (MOFs, CO_2, 298 K, 0.1 bar).

Table S8 Descriptors ranked by mean |SHAP| in the protocol-sensitivity model, with the CRAFTED descriptor name and the direction of the effect: the direction index is the mean SHAP value of the MOFs in the highest fifth of the descriptor minus that of the lowest fifth, divided by the mean |SHAP|; |index| ≥ 0.5 is read as a clear direction.

Table S9 Variance decomposition on all MOFs and on the MOFChecker-clean and flagged subsets (298 K).

Table S10 Cross-code reproduction: CO_2 isotherm of MULQOA (UFF + DDEC, 298 K) from CRAFTED (RASPA2) and from RASPA3 with pyMSER truncation, 10,000 cycles per point.

Table S11 Replicate RASPA3 runs of the 30 sampled MOFs (UFF + DDEC, CO_2, 298 K) and their comparison with CRAFTED.

Table S12 Long-run (40,000-cycle) check of the conditions that disagreed with CRAFTED by more than 2σ after 10,000 cycles, and of the controls.

Table S13 Factor effects measured with RASPA3 on the 30 sampled MOFs, with the CRAFTED variance components and protocol effects at the same condition.

Table S14 Experimental anchor: measured CO_2 uptake from the NIST/ARPA-E database interpolated at 0.1 and 1 bar, against the CRAFTED protocol range at 298 K.

Table S15 Scatter between laboratories and between protocols for the materials measured by more than one group.

Table S16 Structure source: reference-protocol uptake (UFF + DDEC, 298 K) across the CoRE MOF 2014 CIFs of the same composition.

All tables are also supplied as machine-readable CSV files (one file per table, Table_S<n>_<name>.csv, in the ESI tables archive), together with the complete per-MOF sensitivity and factor tables.

S1 Reproducing every result in the article

The deposited code[32] runs on Windows, macOS or Linux with Python 3.10–3.12. Each step is one command, python scripts/run_step.py <step> (or one click in the supplied VS Code workspace), which logs its output and adds a line to results/progress_log.csv.

Step	What it does	Run time
02	Download CRAFTED 2.0.1 (55 MB) from Zenodo	10–30 min
10	MOFChecker validity flags for the 690 MOFs	30–60 min
11, 11c	Cross-code reproduction of the MULQOA isotherm with RASPA3 and comparison with CRAFTED (Fig. 9)	30–60 min
12	Gauge R&R: variance components, bootstrap intervals, REML check, validity subsets (Table 1, Figs 2, 3, 7, 8)	5 min
13	Top-k agreement between protocols (Table 2, Fig. 4)	1 min
14, 14cof	Protocol-sensitivity model and SHAP analysis (Figs 5, 6)	2–5 min
15, 15b	240 RASPA3 runs on 30 MOFs; 40,000-cycle checks of the discrepant conditions	about 150 CPU-hours
16	Factor analysis and re-analysis with the measured repeatability (Tables 3, 4, Figs 10–13)	10 s
17	Experimental anchor against NIST/ARPA-E isotherms (Fig. 14)	1 min, needs internet
18	Reference list checked against Crossref and DataCite	1 min, needs internet
P1	build_paper1.py: all figures, tables, the manuscript, this ESI and the cover letter	15 s
19	Submission package: code-and-data deposit and preflight report	30 s

S2 Settings of the new RASPA3 simulations

• Code: RASPA3 3.1 through its Python interface (scripts/gcmc_raspa3.py), grand-canonical ensemble at 298 K.

• Framework: the CRAFTED CIF with DDEC charges, rigid, replicated so that every perpendicular width of the simulation box exceeds twice the cut-off.

• Force field: UFF Lennard-Jones parameters from the CRAFTED force-field files, Lorentz–Berthelot mixing; 12.8 Å cut-off with shifted potentials and no tail corrections (the CRAFTED convention) or, for the cut-off factor, 12.0 Å truncated with analytic tail corrections.

• Electrostatics: Ewald summation.

• Sorbates: TraPPE CO_2 (three sites) and TraPPE N_2 (two sites with a massless charged centre); EPM2 CO_2 for the sorbate-model factor. Fugacities from the Peng–Robinson equation of state with the critical constants and acentric factors of CO_2 and N_2.

• Moves: swap (insertion/deletion) 0.5, translation 0.3, rotation 0.1, configurational-bias reinsertion 0.1.

• Sampling: 10,000 cycles without a separate initialisation period (40,000 for the long runs and the N_2 sampling factor), loading recorded every cycle and truncated with pyMSER (batch size 5, lowest-local-minimum criterion); the reported uncertainty is pyMSER's uncorrelated standard deviation.

• Seeds: seeds 1 and 2 for the replicate pairs, seed 3 for the long runs.

Table S1

Variance components for CO_2 uptake of MOFs at 298 K and 0.1 bar by the method of moments and by REML (statsmodels MixedLM).

Estimator	sd material	sd protocol	sd interaction	sd MC	%GRR	NDC
REML	0.8664	0.3282	0.4604	0.1574	56.09	2
Method of moments	0.8664	0.3281	0.4604	0.1574	56.08	2

Table S2

Software environment of the analysis (captured when the manuscript was built).

Component	Version
Python	3.12.5
Operating system	Windows 11
Logical CPU cores	16
numpy	2.5.3
pandas	3.0.6
scipy	1.18.1
statsmodels	0.15.0
scikit-learn	1.9.1
xgboost	3.4.1
shap	0.52.0
matplotlib	3.11.2
pymser	1.0.22
python-docx	1.2.0
mofchecker-next (separate environment)	0.1.2

Table S3

Variance components (sd, ln units), %GRR and NDC with bootstrap 95% confidence intervals for every class, temperature, response and pressure.

Class	T / K	Response	P / bar	n	sd material	sd protocol	sd interaction	sd MC	%GRR	%GRR 95% CI	NDC	NDC 95% CI
MOF	273	CO₂	0.1	660	0.82	0.297	0.435	0.094	54.6	52.0–57.3	2	2–2
MOF	273	CO₂	1	673	0.629	0.135	0.232	0.033	39.5	36.8–42.5	3	2–3
MOF	273	CO₂	10	681	0.778	0.054	0.131	0.018	18.1	15.0–21.0	7	6–9
MOF	273	N₂	0.1	279	0.54	0.19	0.0	0.53	72.2	66.3–79.1	1	1–1
MOF	273	N₂	1	634	0.578	0.112	0.151	0.214	44.2	41.0–47.3	2	2–3
MOF	273	N₂	10	666	0.623	0.081	0.16	0.074	29.8	27.7–32.0	4	4–4
MOF	298	CO₂	0.1	632	0.866	0.328	0.46	0.157	56.1	53.4–58.8	2	1–2
MOF	298	CO₂	1	669	0.614	0.176	0.284	0.053	48.3	46.0–50.9	2	2–2
MOF	298	CO₂	10	677	0.717	0.071	0.145	0.024	22.2	19.1–25.2	6	5–7
MOF	298	N₂	0.1	99	0.584	0.244	0.0	0.649	76.5	66.6–89.1	1	0–1
MOF	298	N₂	1	614	0.546	0.107	0.0	0.29	49.3	47.0–51.6	2	2–2
MOF	298	N₂	10	659	0.611	0.082	0.153	0.099	31.2	28.8–33.9	4	3–4
MOF	323	CO₂	0.1	553	0.798	0.346	0.452	0.222	60.8	57.7–63.6	1	1–1
MOF	323	CO₂	1	662	0.622	0.209	0.321	0.08	53.3	50.7–55.9	2	2–2
MOF	323	CO₂	10	677	0.684	0.091	0.184	0.031	29.1	25.9–32.9	4	4–5
MOF	323	N₂	1	600	0.53	0.102	0.0	0.367	58.4	55.6–61.3	1	1–2
MOF	323	N₂	10	651	0.593	0.082	0.134	0.126	32.1	29.4–34.8	4	3–4
MOF	273	CO₂/N₂ selectivity		630	0.441	0.15	0.196	0.227	60.5	57.5–63.6	1	1–2
MOF	298	CO₂/N₂ selectivity		613	0.498	0.188	0.0	0.316	59.4	57.3–62.5	1	1–2
MOF	323	CO₂/N₂ selectivity		598	0.577	0.212	0.0	0.41	62.5	60.6–65.0	1	1–1
COF	273	CO₂	0.1	565	0.934	0.448	0.247	0.472	59.8	56.8–63.2	1	1–2
COF	273	CO₂	1	663	0.669	0.359	0.322	0.162	60.5	57.7–63.3	1	1–1
COF	273	CO₂	10	664	0.49	0.168	0.137	0.053	41.4	38.2–45.5	3	2–3
COF	273	N₂	0.1	62	0.355	0.224	0.0	0.842	92.6	89.8–95.2	0	0–0
COF	273	N₂	1	660	0.494	0.187	0.0	0.468	71.4	67.9–74.6	1	1–1
COF	273	N₂	10	662	0.422	0.149	0.0	0.145	44.2	41.4–47.2	2	2–3
COF	298	CO₂	0.1	334	0.865	0.387	0.0	0.508	59.4	55.7–63.5	1	1–2
COF	298	CO₂	1	663	0.678	0.342	0.303	0.22	60.0	57.0–62.9	1	1–2
COF	298	CO₂	10	663	0.455	0.205	0.159	0.072	50.9	47.7–54.1	2	2–2
COF	298	N₂	1	655	0.458	0.166	0.0	0.592	80.2	78.0–82.2	1	0–1
COF	298	N₂	10	662	0.429	0.139	0.0	0.169	45.4	42.8–48.3	2	2–2
COF	323	CO₂	0.1	200	0.766	0.341	0.0	0.575	65.7	61.5–71.3	1	1–1
COF	323	CO₂	1	660	0.653	0.318	0.23	0.291	59.8	56.9–62.6	1	1–2
COF	323	CO₂	10	663	0.459	0.217	0.169	0.094	53.6	50.4–56.7	2	2–2
COF	323	N₂	1	645	0.428	0.148	0.0	0.683	85.3	83.7–87.0	0	0–0

COF	323	N_2	10	662	0.437	0.128	0.0	0.189	46.4	43.6–49.7	2	2–2
COF	273	CO_2/N_2 selectivity		656	0.63	0.285	0.0	0.604	72.8	70.8–75.2	1	1–1
COF	298	CO_2/N_2 selectivity		629	0.625	0.263	0.0	0.803	80.4	78.4–82.3	1	0–1
COF	323	CO_2/N_2 selectivity		516	0.588	0.227	0.0	0.945	85.6	83.9–87.2	0	0–0

Table S4

Protocol main effects at 298 K: mean change of ln(uptake) relative to UFF + DDEC, the corresponding factor on the uptake and its sd across materials.

Class	Response	P / bar	Protocol	Mean Δ ln u vs UFF + DDEC	Factor	sd across materials	n
MOF	CO_2	0.1	UFF + no charges	-0.751	0.47	0.673	632
MOF	CO_2	0.1	UFF + Qeq	0.127	1.13	0.703	632
MOF	CO_2	0.1	UFF + EQeq	0.028	1.03	0.649	632
MOF	CO_2	0.1	UFF + PACMOF	-0.008	0.99	0.271	632
MOF	CO_2	0.1	UFF + MPNN	0.037	1.04	0.277	632
MOF	CO_2	0.1	UFF + DDEC	0.0	1.0	0.0	632
MOF	CO_2	0.1	DREIDING + no charges	-0.931	0.39	0.775	632
MOF	CO_2	0.1	DREIDING + Qeq	-0.0	1.0	0.804	632
MOF	CO_2	0.1	DREIDING + EQeq	-0.332	0.72	0.671	632
MOF	CO_2	0.1	DREIDING + PACMOF	-0.245	0.78	0.522	632
MOF	CO_2	0.1	DREIDING + MPNN	-0.212	0.81	0.518	632
MOF	CO_2	0.1	DREIDING + DDEC	-0.24	0.79	0.462	632
MOF	CO_2	1	UFF + no charges	-0.388	0.68	0.427	669
MOF	CO_2	1	UFF + Qeq	0.046	1.05	0.326	669
MOF	CO_2	1	UFF + EQeq	0.006	1.01	0.354	669
MOF	CO_2	1	UFF + PACMOF	0.004	1.0	0.192	669
MOF	CO_2	1	UFF + MPNN	0.018	1.02	0.164	669
MOF	CO_2	1	UFF + DDEC	0.0	1.0	0.0	669
MOF	CO_2	1	DREIDING + no charges	-0.526	0.59	0.523	669
MOF	CO_2	1	DREIDING + Qeq	-0.06	0.94	0.435	669
MOF	CO_2	1	DREIDING + EQeq	-0.196	0.82	0.437	669
MOF	CO_2	1	DREIDING + PACMOF	-0.163	0.85	0.351	669
MOF	CO_2	1	DREIDING + MPNN	-0.152	0.86	0.309	669
MOF	CO_2	1	DREIDING + DDEC	-0.168	0.85	0.294	669
MOF	CO_2	10	UFF + no charges	-0.122	0.88	0.212	677
MOF	CO_2	10	UFF + Qeq	0.033	1.03	0.177	677
MOF	CO_2	10	UFF + EQeq	0.004	1.0	0.148	677
MOF	CO_2	10	UFF + PACMOF	-0.0	1.0	0.08	677
MOF	CO_2	10	UFF + MPNN	0.004	1.0	0.048	677
MOF	CO_2	10	UFF + DDEC	0.0	1.0	0.0	677
MOF	CO_2	10	DREIDING + no charges	-0.21	0.81	0.251	677
MOF	CO_2	10	DREIDING + Qeq	-0.05	0.95	0.232	677
MOF	CO_2	10	DREIDING + EQeq	-0.099	0.91	0.192	677
MOF	CO_2	10	DREIDING + PACMOF	-0.098	0.91	0.189	677
MOF	CO_2	10	DREIDING + MPNN	-0.092	0.91	0.189	677
MOF	CO_2	10	DREIDING + DDEC	-0.096	0.91	0.163	677
MOF	N_2	0.1	UFF + no charges	-0.116	0.89	0.17	99

MOF	N_2	0.1	UFF + Qeq	0.514	1.67	1.264	99
MOF	N_2	0.1	UFF + EQeq	0.036	1.04	0.207	99
MOF	N_2	0.1	UFF + PACMOF	0.004	1.0	0.114	99
MOF	N_2	0.1	UFF + MPNN	0.009	1.01	0.053	99
MOF	N_2	0.1	UFF + DDEC	0.0	1.0	0.0	99
MOF	N_2	0.1	DREIDING + no charges	-0.343	0.71	0.287	99
MOF	N_2	0.1	DREIDING + Qeq	0.288	1.33	1.322	99
MOF	N_2	0.1	DREIDING + EQeq	-0.232	0.79	0.264	99
MOF	N_2	0.1	DREIDING + PACMOF	-0.241	0.79	0.299	99
MOF	N_2	0.1	DREIDING + MPNN	-0.24	0.79	0.28	99
MOF	N_2	0.1	DREIDING + DDEC	-0.25	0.78	0.268	99
MOF	N_2	1	UFF + no charges	-0.126	0.88	0.18	614
MOF	N_2	1	UFF + Qeq	0.107	1.11	0.456	614
MOF	N_2	1	UFF + EQeq	0.025	1.02	0.152	614
MOF	N_2	1	UFF + PACMOF	-0.004	1.0	0.064	614
MOF	N_2	1	UFF + MPNN	0.004	1.0	0.054	614
MOF	N_2	1	UFF + DDEC	0.0	1.0	0.0	614
MOF	N_2	1	DREIDING + no charges	-0.268	0.76	0.357	614
MOF	N_2	1	DREIDING + Qeq	-0.03	0.97	0.537	614
MOF	N_2	1	DREIDING + EQeq	-0.158	0.85	0.33	614
MOF	N_2	1	DREIDING + PACMOF	-0.157	0.85	0.325	614
MOF	N_2	1	DREIDING + MPNN	-0.153	0.86	0.324	614
MOF	N_2	1	DREIDING + DDEC	-0.155	0.86	0.315	614
MOF	N_2	10	UFF + no charges	-0.087	0.92	0.139	659
MOF	N_2	10	UFF + Qeq	0.054	1.06	0.244	659
MOF	N_2	10	UFF + EQeq	0.008	1.01	0.109	659
MOF	N_2	10	UFF + PACMOF	-0.002	1.0	0.047	659
MOF	N_2	10	UFF + MPNN	0.004	1.0	0.041	659
MOF	N_2	10	UFF + DDEC	0.0	1.0	0.0	659
MOF	N_2	10	DREIDING + no charges	-0.214	0.81	0.317	659
MOF	N_2	10	DREIDING + Qeq	-0.06	0.94	0.359	659
MOF	N_2	10	DREIDING + EQeq	-0.141	0.87	0.287	659
MOF	N_2	10	DREIDING + PACMOF	-0.136	0.87	0.275	659
MOF	N_2	10	DREIDING + MPNN	-0.131	0.88	0.269	659
MOF	N_2	10	DREIDING + DDEC	-0.135	0.87	0.266	659
MOF	CO_2/N_2 selectivity		UFF + no charges	-0.488	0.61	0.466	613
MOF	CO_2/N_2 selectivity		UFF + Qeq	0.003	1.0	0.479	613
MOF	CO_2/N_2 selectivity		UFF + EQeq	0.02	1.02	0.459	613
MOF	CO_2/N_2 selectivity		UFF + PACMOF	-0.005	1.0	0.189	613
MOF	CO_2/N_2 selectivity		UFF + MPNN	0.026	1.03	0.213	613
MOF	CO_2/N_2 selectivity		UFF + DDEC	0.0	1.0	0.0	613
MOF	CO_2/N_2 selectivity		DREIDING + no charges	-0.49	0.61	0.482	613
MOF	CO_2/N_2 selectivity		DREIDING + Qeq	0.038	1.04	0.505	613
MOF	CO_2/N_2 selectivity		DREIDING + EQeq	-0.093	0.91	0.381	613
MOF	CO_2/N_2 selectivity		DREIDING + PACMOF	-0.052	0.95	0.299	613
MOF	CO_2/N_2 selectivity		DREIDING + MPNN	-0.03	0.97	0.285	613

MOF	CO_2/N_2 selectivity		DREIDING + DDEC	-0.053	0.95	0.244	613
COF	CO_2	0.1	UFF + no charges	-0.309	0.73	0.489	334
COF	CO_2	0.1	UFF + Qeq	0.261	1.3	0.825	334
COF	CO_2	0.1	UFF + EQeq	-0.162	0.85	0.438	334
COF	CO_2	0.1	UFF + PACMOF	0.202	1.22	0.379	334
COF	CO_2	0.1	UFF + MPNN	0.059	1.06	0.28	334
COF	CO_2	0.1	UFF + DDEC	0.0	1.0	0.0	334
COF	CO_2	0.1	DREIDING + no charges	-0.941	0.39	0.489	334
COF	CO_2	0.1	DREIDING + Qeq	-0.338	0.71	0.922	334
COF	CO_2	0.1	DREIDING + EQeq	-0.797	0.45	0.488	334
COF	CO_2	0.1	DREIDING + PACMOF	-0.413	0.66	0.467	334
COF	CO_2	0.1	DREIDING + MPNN	-0.565	0.57	0.378	334
COF	CO_2	0.1	DREIDING + DDEC	-0.626	0.53	0.255	334
COF	CO_2	1	UFF + no charges	-0.277	0.76	0.496	663
COF	CO_2	1	UFF + Qeq	0.233	1.26	0.627	663
COF	CO_2	1	UFF + EQeq	-0.159	0.85	0.43	663
COF	CO_2	1	UFF + PACMOF	0.222	1.25	0.314	663
COF	CO_2	1	UFF + MPNN	0.065	1.07	0.213	663
COF	CO_2	1	UFF + DDEC	0.0	1.0	0.0	663
COF	CO_2	1	DREIDING + no charges	-0.828	0.44	0.462	663
COF	CO_2	1	DREIDING + Qeq	-0.261	0.77	0.719	663
COF	CO_2	1	DREIDING + EQeq	-0.705	0.49	0.438	663
COF	CO_2	1	DREIDING + PACMOF	-0.291	0.75	0.402	663
COF	CO_2	1	DREIDING + MPNN	-0.464	0.63	0.313	663
COF	CO_2	1	DREIDING + DDEC	-0.527	0.59	0.213	663
COF	CO_2	10	UFF + no charges	-0.111	0.9	0.202	663
COF	CO_2	10	UFF + Qeq	0.1	1.11	0.24	663
COF	CO_2	10	UFF + EQeq	-0.062	0.94	0.172	663
COF	CO_2	10	UFF + PACMOF	0.1	1.11	0.125	663
COF	CO_2	10	UFF + MPNN	0.042	1.04	0.082	663
COF	CO_2	10	UFF + DDEC	0.0	1.0	0.0	663
COF	CO_2	10	DREIDING + no charges	-0.498	0.61	0.226	663
COF	CO_2	10	DREIDING + Qeq	-0.214	0.81	0.33	663
COF	CO_2	10	DREIDING + EQeq	-0.43	0.65	0.224	663
COF	CO_2	10	DREIDING + PACMOF	-0.218	0.8	0.21	663
COF	CO_2	10	DREIDING + MPNN	-0.298	0.74	0.181	663
COF	CO_2	10	DREIDING + DDEC	-0.35	0.7	0.168	663
COF	N_2	1	UFF + no charges	-0.028	0.97	0.054	655
COF	N_2	1	UFF + Qeq	0.049	1.05	0.147	655
COF	N_2	1	UFF + EQeq	-0.015	0.98	0.054	655
COF	N_2	1	UFF + PACMOF	0.027	1.03	0.053	655
COF	N_2	1	UFF + MPNN	0.007	1.01	0.033	655
COF	N_2	1	UFF + DDEC	0.0	1.0	0.0	655
COF	N_2	1	DREIDING + no charges	-0.341	0.71	0.14	655
COF	N_2	1	DREIDING + Qeq	-0.266	0.77	0.204	655
COF	N_2	1	DREIDING + EQeq	-0.328	0.72	0.147	655
COF	N_2	1	DREIDING + PACMOF	-0.286	0.75	0.15	655
COF	N_2	1	DREIDING + MPNN	-0.306	0.74	0.145	655
COF	N_2	1	DREIDING +	-0.313	0.73	0.142	655

			DDEC				
COF	N_2	10	UFF + no charges	-0.023	0.98	0.041	662
COF	N_2	10	UFF + Qeq	0.041	1.04	0.126	662
COF	N_2	10	UFF + EQeq	-0.011	0.99	0.041	662
COF	N_2	10	UFF + PACMOF	0.022	1.02	0.041	662
COF	N_2	10	UFF + MPNN	0.007	1.01	0.025	662
COF	N_2	10	UFF + DDEC	0.0	1.0	0.0	662
COF	N_2	10	DREIDING + no charges	-0.285	0.75	0.101	662
COF	N_2	10	DREIDING + Qeq	-0.223	0.8	0.155	662
COF	N_2	10	DREIDING + EQeq	-0.275	0.76	0.107	662
COF	N_2	10	DREIDING + PACMOF	-0.239	0.79	0.112	662
COF	N_2	10	DREIDING + MPNN	-0.256	0.77	0.107	662
COF	N_2	10	DREIDING + DDEC	-0.262	0.77	0.105	662
COF	CO_2/N_2 selectivity		UFF + no charges	-0.309	0.73	0.582	629
COF	CO_2/N_2 selectivity		UFF + Qeq	0.269	1.31	0.745	629
COF	CO_2/N_2 selectivity		UFF + EQeq	-0.181	0.83	0.513	629
COF	CO_2/N_2 selectivity		UFF + PACMOF	0.253	1.29	0.388	629
COF	CO_2/N_2 selectivity		UFF + MPNN	0.052	1.05	0.29	629
COF	CO_2/N_2 selectivity		UFF + DDEC	0.0	1.0	0.0	629
COF	CO_2/N_2 selectivity		DREIDING + no charges	-0.582	0.56	0.558	629
COF	CO_2/N_2 selectivity		DREIDING + Qeq	0.027	1.03	0.804	629
COF	CO_2/N_2 selectivity		DREIDING + EQeq	-0.453	0.64	0.521	629
COF	CO_2/N_2 selectivity		DREIDING + PACMOF	0.003	1.0	0.442	629
COF	CO_2/N_2 selectivity		DREIDING + MPNN	-0.211	0.81	0.343	629
COF	CO_2/N_2 selectivity		DREIDING + DDEC	-0.263	0.77	0.136	629

Table S5

Agreement of the top-k short-lists of the 12 protocols (MOFs) for every temperature, response, pressure and k.

T / K	Response	P / bar	k	n	Mean Jaccard	Min Jaccard	Reference top-k kept (mean)	Reference top-k kept (min)	Least similar protocol	In all 12	Mean Spearman
298	CO_2	0.1	10	632	0.173	0.0	3.0	0	UFF + no charges	0	0.763
298	CO_2	0.1	25	632	0.243	0.0	11.3	4	UFF + no charges	0	0.763
298	CO_2	0.1	50	632	0.303	0.053	25.8	11	DREIDING + no charges	4	0.763
298	CO_2	0.1	100	632	0.401	0.124	62.0	40	DREIDING + no charges	10	0.763
298	CO_2	1	10	669	0.286	0.0	4.9	1	DREIDING + Qeq	0	0.86
298	CO_2	1	25	669	0.352	0.087	14.6	7	DREIDING + no charges	1	0.86
298	CO_2	1	50	669	0.446	0.22	34.1	24	UFF + no charges	7	0.86
298	CO_2	1	100	669	0.529	0.258	74.0	57	DREIDING + no charges	30	0.86
298	CO_2	10	10	677	0.407	0.176	6.5	5	DREIDING + no charges	1	0.981
298	CO_2	10	25	677	0.508	0.25	18.6	12	DREIDING + Qeq	5	0.981
298	CO_2	10	50	677	0.65	0.449	40.2	34	UFF +	22	0.981

									EQeq		
298	CO_2	10	100	677	0.829	0.739	91.3	87	UFF + Qeq	71	0.981
273	CO_2	0.1	10	660	0.198	0.0	3.6	0	UFF + no charges	0	0.774
273	CO_2	0.1	25	660	0.249	0.02	11.1	2	UFF + no charges	0	0.774
273	CO_2	0.1	50	660	0.348	0.042	28.8	8	DREIDING + no charges	2	0.774
273	CO_2	0.1	100	660	0.419	0.13	65.5	36	DREIDING + no charges	8	0.774
323	CO_2	0.1	10	553	0.275	0.0	4.5	0	UFF + Qeq	0	0.74
323	CO_2	0.1	25	553	0.282	0.064	12.4	4	UFF + Qeq	3	0.74
323	CO_2	0.1	50	553	0.336	0.087	28.5	16	UFF + no charges	6	0.74
323	CO_2	0.1	100	553	0.406	0.149	63.3	38	UFF + no charges	12	0.74
298	N_2	1	10	614	0.538	0.053	7.5	1	UFF + Qeq	1	0.894
298	N_2	1	25	614	0.47	0.136	17.6	6	UFF + Qeq	6	0.894
298	N_2	1	50	614	0.488	0.19	35.3	20	DREIDING + Qeq	13	0.894
298	N_2	1	100	614	0.639	0.399	79.3	62	DREIDING + Qeq	43	0.894
298	CO_2/N_2 selectivity		10	613	0.286	0.0	5.0	1	UFF + no charges	0	0.694
298	CO_2/N_2 selectivity		25	613	0.27	0.02	10.9	3	UFF + no charges	1	0.694
298	CO_2/N_2 selectivity		50	613	0.308	0.031	24.1	13	DREIDING + no charges	1	0.694
298	CO_2/N_2 selectivity		100	613	0.421	0.136	64.2	37	UFF + no charges	11	0.694
298	CO_2	0.1	10	334	0.3	0.053	4.8	1	UFF + Qeq	1	0.815
298	CO_2	0.1	25	334	0.459	0.19	17.1	10	UFF + Qeq	6	0.815
298	CO_2	0.1	50	334	0.58	0.389	38.9	30	DREIDING + Qeq	22	0.815
298	CO_2	0.1	100	334	0.679	0.439	83.7	70	DREIDING + Qeq	53	0.815
298	CO_2	1	10	663	0.307	0.111	5.0	2	UFF + Qeq	2	0.772
298	CO_2	1	25	663	0.381	0.087	15.0	4	DREIDING + Qeq	4	0.772
298	CO_2	1	50	663	0.427	0.176	33.0	15	DREIDING + Qeq	13	0.772
298	CO_2	1	100	663	0.5	0.258	69.9	49	DREIDING + Qeq	30	0.772
298	CO_2	10	10	663	0.492	0.111	7.0	3	DREIDING + Qeq	1	0.831
298	CO_2	10	25	663	0.636	0.429	20.2	15	UFF + Qeq	14	0.831
298	CO_2	10	50	663	0.725	0.515	42.4	35	DREIDING + Qeq	32	0.831
298	CO_2	10	100	663	0.714	0.493	85.4	69	DREIDING + Qeq	64	0.831
273	CO_2	0.1	10	565	0.311	0.053	5.5	1	DREIDING + Qeq	1	0.765
273	CO_2	0.1	25	565	0.368	0.087	15.1	4	UFF + Qeq	3	0.765
273	CO_2	0.1	50	565	0.422	0.205	30.7	19	UFF + Qeq	14	0.765
273	CO_2	0.1	100	565	0.516	0.25	72.0	52	DREIDING + Qeq	33	0.765
323	CO_2	0.1	10	200	0.377	0.111	5.7	4	DREIDING + Qeq	2	0.861
323	CO_2	0.1	25	200	0.547	0.389	17.9	16	DREIDING + PACMOF	9	0.861
323	CO_2	0.1	50	200	0.677	0.538	41.8	36	DREIDING + Qeq	29	0.861
323	CO_2	0.1	100	200	0.783	0.613	89.7	83	DREIDING + Qeq	68	0.861
298	N_2	1	10	655	0.628	0.333	8.1	7	DREIDING + no charges	4	0.932
298	N_2	1	25	655	0.695	0.515	20.5	17	DREIDING + no charges	14	0.932
298	N_2	1	50	655	0.736	0.562	42.2	37	DREIDING + Qeq	32	0.932

298	N_2	1	100	655	0.786	0.639	88.3	79	DREIDING + Qeq	74	0.932
298	CO_2/N_2 selectivity		10	629	0.138	0.0	2.7	0	UFF + Qeq	0	0.784
298	CO_2/N_2 selectivity		25	629	0.199	0.0	8.2	2	UFF + Qeq	0	0.784
298	CO_2/N_2 selectivity		50	629	0.311	0.075	24.5	14	UFF + no charges	4	0.784
298	CO_2/N_2 selectivity		100	629	0.426	0.149	65.4	48	UFF + Qeq	18	0.784

Table S6

Per-MOF protocol sensitivity (CO_2, 298 K, 0.1 bar) of the 20 most and the 20 least sensitive MOFs; all MOFs are in the CSV file.

MOF	Sensitivity (interaction sd)	Charge-scheme sd (UFF)	UFF–DREIDING gap	ln uptake, UFF + DDEC	max/min
LURGEL	1.532	1.717	0.242	-0.769	143.06
CUYQIX	1.251	0.609	2.314	0.488	46.98
XINWUO	1.17	1.484	0.899	-2.067	76.63
SENWIT	1.164	1.634	0.878	-1.758	62.5
PARHAS	1.152	1.371	0.341	1.768	97.85
EHALOP	1.099	1.11	0.567	0.274	33.76
SENWEP	1.081	1.53	0.41	-0.544	38.15
EGELUY01	1.075	1.12	0.576	0.238	32.17
NARTUX	1.066	1.192	0.409	0.629	48.21
QONQEQ	1.061	1.092	0.584	0.255	33.93
ITETEH	1.055	1.27	0.842	-0.119	44.52
SENWAL	1.053	1.519	0.902	-2.01	48.57
KIGCEK	1.047	1.242	1.287	-1.785	50.57
BEPPOD	1.04	0.952	1.823	-0.347	51.61
BEPMAM	1.039	1.298	1.186	-2.23	52.8
BEPLUF	1.034	1.273	1.207	-2.155	53.08
SENWOZ	1.028	1.49	0.882	-1.956	47.56
WAYMIU	1.023	1.017	0.581	0.494	27.63
BEPPIX	1.022	1.125	1.464	-1.719	52.22
SABVUN	1.02	1.045	0.585	0.456	29.36
LOWPUI	0.082	0.385	0.246	0.953	2.9
TASXIW	0.095	0.326	0.143	-0.178	2.76
LEGGEK	0.114	0.303	0.269	1.084	3.2
UXUZAP	0.117	0.246	0.167	0.841	2.26
NAQLAT	0.123	0.213	0.174	0.75	2.22
MEJQID	0.127	0.249	0.174	0.764	2.23
MEJQOJ	0.13	0.238	0.169	0.725	2.18
BENXUP	0.134	0.355	0.239	0.717	4.04
MAZSUD	0.139	0.324	0.078	0.172	2.65
FIPWUY	0.14	0.206	0.229	1.14	2.49
XAXQEU	0.144	0.334	0.091	1.17	2.71
MEJQEZ	0.144	0.261	0.144	0.919	2.35
RUMWUS	0.144	0.261	0.386	1.011	2.93
KIXXOG	0.149	0.155	0.124	1.436	2.28
HIWLOO	0.149	0.219	0.018	0.186	1.93
RETBEZ	0.151	0.355	0.316	0.664	3.56
KANDIO	0.155	0.257	0.28	0.424	2.99
KIDDOS	0.157	0.502	0.162	1.293	3.49
PURJIW	0.159	0.349	0.429	-0.283	3.11
MIZJUB	0.16	0.387	0.393	-0.422	3.82

Table S7

Five-fold cross-validated performance of the gradient-boosting models of protocol sensitivity (MOFs, CO_2, 298 K, 0.1 bar).

Target	n	Cross-validated R^2	Mean	sd

Protocol sensitivity (12 protocols)	632	0.427	0.428	0.231
Charge-scheme spread (UFF)	632	0.485	0.472	0.311
UFF–DREIDING gap	632	0.496	0.404	0.315
ln uptake, reference protocol	632	0.751	-0.09	1.007

Table S8

Descriptors ranked by mean |SHAP| in the protocol-sensitivity model, with the CRAFTED descriptor name and the direction of the effect: the direction index is the mean SHAP value of the MOFs in the highest fifth of the descriptor minus that of the lowest fifth, divided by the mean |SHAP|; |index| ≥ 0.5 is read as a clear direction.

Rank	Descriptor	CRAFTED name	Mean |SHAP|	Direction index	Direction
1	Δ atomic number · metal centre · depth 1	D_mc-Z-1-all	0.0264	-2.2	low values raise sensitivity
2	largest included sphere (Å)	D_is	0.0113	-1.58	low values raise sensitivity
3	coordination number · functional group · depth 2	func-T-2-all	0.0099	-1.84	low values raise sensitivity
4	unit-cell volume (Å^3)	Unitcell_volume	0.0094	-1.16	low values raise sensitivity
5	atomic number · metal centre · depth 1	mc-Z-1-all	0.0094	-3.43	low values raise sensitivity
6	Δ polarizability · linker-connecting atom · depth 3	D_lc-alpha-3-all	0.0089	1.98	high values raise sensitivity
7	Δ atomic number · metal centre · depth 2	D_mc-Z-2-all	0.0088	-2.73	low values raise sensitivity
8	Δ electronegativity · linker-connecting atom · depth 3	D_lc-chi-3-all	0.0083	2.36	high values raise sensitivity
9	electronegativity · linker-connecting atom · depth 3	lc-chi-3-all	0.0072	2.26	high values raise sensitivity
10	electronegativity · metal centre · depth 3	mc-chi-3-all	0.0071	0.67	high values raise sensitivity
11	accessible surface area (m^2/g)	ASA_m^2/g	0.0069	1.66	high values raise sensitivity
12	electronegativity · metal centre · depth 2	mc-chi-2-all	0.0069	1.18	high values raise sensitivity
13	accessible surface area (m^2/cm^3)	ASA_m^2/cm^3	0.0067	2.65	high values raise sensitivity
14	density (g/cm^3)	Density	0.0063	1.89	high values raise sensitivity
15	included sphere along the free path (Å)	D_isfs	0.0061	-2.16	low values raise sensitivity
16	atomic number · metal centre · depth 2	mc-Z-2-all	0.0058	-1.18	low values raise sensitivity
17	identity · linker-connecting atom · depth 3	lc-I-3-all	0.0058	2.23	high values raise sensitivity
18	largest free sphere (Å)	D_fs	0.0057	-1.69	low values raise sensitivity
19	electronegativity · whole linker · depth 1	f-lig-chi-1	0.0054	-2.76	low values raise sensitivity
20	electronegativity · metal centre · depth 1	mc-chi-1-all	0.0053	-2.56	low values raise sensitivity
21	Δ coordination number · metal centre · depth 3	D_mc-T-3-all	0.0051		
22	covalent radius · whole structure · depth 0	f-S-0-all	0.0051		
23	Δ covalent radius · linker-connecting atom · depth 3	D_lc-S-3-all	0.0049		
24	accessible void fraction	AV_Volume_fraction	0.0048		
25	coordination number · metal centre · depth 3	mc-T-3-all	0.0043		
26	Δ covalent radius · functional group · depth 2	D_func-S-2-all	0.0042		

27	covalent radius · metal centre · depth 3	mc-S-3-all	0.0041		
28	covalent radius · linker-connecting atom · depth 3	lc-S-3-all	0.0039		
29	coordination number · whole linker · depth 2	f-lig-T-2	0.0039		
30	Δ atomic number · functional group · depth 1	D_func-Z-1-all	0.0038		

Table S9

Variance decomposition on all MOFs and on the MOFChecker-clean and flagged subsets (298 K).

T / K	Response	P / bar	Subset	n	%GRR	95% CI	NDC	sd interaction
298	CO₂	0.1	all	632	56.1	53.6–59.0	2	0.46
298	CO₂	0.1	clean	550	55.3	52.7–58.4	2	0.438
298	CO₂	0.1	flagged	82	61.0	53.2–68.8	1	0.572
298	CO₂	1	all	669	48.3	45.8–50.8	2	0.284
298	CO₂	1	clean	580	47.3	44.9–50.0	2	0.272
298	CO₂	1	flagged	89	52.6	46.8–59.3	2	0.349
298	CO₂	10	all	677	22.2	19.5–25.5	6	0.145
298	CO₂	10	clean	587	22.3	18.9–25.9	6	0.144
298	CO₂	10	flagged	90	21.2	18.5–25.9	6	0.143
298	CO₂/N₂ selectivity		all	613	59.4	56.9–62.2	1	0.0
298	CO₂/N₂ selectivity		clean	538	60.7	58.5–63.6	1	0.0
298	CO₂/N₂ selectivity		flagged	75	60.7	53.0–70.0	1	0.259

Table S10

Cross-code reproduction: CO₂ isotherm of MULQOA (UFF + DDEC, 298 K) from CRAFTED (RASPA2) and from RASPA3 with pyMSER truncation, 10,000 cycles per point.

P / bar	CRAFTED / mol kg⁻¹	RASPA3 / mol kg⁻¹	z	Relative difference / %	MSER cut / cycles
0.001	0.108 ± 0.041	0.111 ± 0.043	0.05	2.8	20
0.002	0.206 ± 0.057	0.213 ± 0.056	0.08	3.2	30
0.005	0.469 ± 0.088	0.474 ± 0.092	0.04	1.0	455
0.01	0.733 ± 0.107	0.720 ± 0.114	-0.08	-1.8	725
0.02	1.227 ± 0.103	1.117 ± 0.089	-0.81	-9.0	1005
0.05	1.978 ± 0.120	1.952 ± 0.139	-0.14	-1.3	970
0.1	2.624 ± 0.145	2.651 ± 0.149	0.13	1.0	635
0.2	3.573 ± 0.167	3.529 ± 0.183	-0.18	-1.2	735
0.5	5.004 ± 0.160	4.933 ± 0.186	-0.29	-1.4	790
1	5.948 ± 0.139	5.915 ± 0.142	-0.16	-0.6	1020
2	6.608 ± 0.126	6.582 ± 0.109	-0.16	-0.4	1190
5	7.372 ± 0.111	7.251 ± 0.108	-0.78	-1.6	1470
10	7.876 ± 0.126	7.614 ± 0.179	-1.19	-3.3	1885

Table S11

Replicate RASPA3 runs of the 30 sampled MOFs (UFF + DDEC, CO₂, 298 K) and their comparison with CRAFTED.

MOF	Group	P / bar	Seed 1 / mol kg⁻¹	Seed 2 / mol kg⁻¹	CRAFTED / mol kg⁻¹	Δ ln u (seeds)	Code effect (ln)	z vs CRAFTED
BENXUP	least sensitive	0.1	2.100 ± 0.109	2.020 ± 0.152	2.048 ± 0.149	0.038	0.006	0.07
BUVXOG	random middle	0.1	0.160 ± 0.074	0.164 ± 0.071	0.163 ± 0.076	-0.028	-0.005	-0.01
CUYQIX	most sensitive	0.1	1.217 ± 0.122	1.516 ± 0.220	1.629 ± 0.097	-0.22	-0.176	-1.65
EGELUY01	most sensitive	0.1	1.252 ± 0.231	1.229 ± 0.231	1.268 ± 0.237	0.018	-0.022	-0.1
EHALOP	most sensitive	0.1	1.322 ± 0.239	1.303 ± 0.238	1.315 ± 0.240	0.015	-0.002	-0.01

FIPWUY	least sensitive	0.1	3.141 ± 0.108	3.172 ± 0.098	3.127 ± 0.091	-0.01	0.009	0.25
KAXQIL	random middle	0.1	1.310 ± 0.076	1.278 ± 0.085	1.317 ± 0.060	0.025	-0.018	-0.28
LEGGEK	least sensitive	0.1	2.791 ± 0.151	2.920 ± 0.119	2.957 ± 0.090	-0.045	-0.035	-0.77
LOWPUI	least sensitive	0.1	2.651 ± 0.301	2.623 ± 0.218	2.594 ± 0.235	0.011	0.016	0.14
LURGEL	most sensitive	0.1	0.478 ± 0.158	0.465 ± 0.159	0.463 ± 0.169	0.026	0.017	0.04
MAZSUD	least sensitive	0.1	1.199 ± 0.195	1.130 ± 0.206	1.187 ± 0.194	0.059	-0.019	-0.09
MEJQID	least sensitive	0.1	2.216 ± 0.171	2.115 ± 0.169	2.147 ± 0.179	0.047	0.008	0.08
MEJQOJ	least sensitive	0.1	2.104 ± 0.215	2.072 ± 0.156	2.066 ± 0.191	0.016	0.011	0.1
NAQLAT	least sensitive	0.1	2.129 ± 0.118	2.055 ± 0.135	2.116 ± 0.132	0.036	-0.011	-0.15
NARTUX	most sensitive	0.1	1.163 ± 0.149	1.336 ± 0.038	1.876 ± 0.076	-0.139	-0.407	-5.8
NISCOJ	random middle	0.1	1.248 ± 0.144	1.171 ± 0.121	1.248 ± 0.115	0.064	-0.031	-0.26
OGAGIN	random middle	0.1	1.462 ± 0.124	1.473 ± 0.106	1.476 ± 0.115	-0.008	-0.006	-0.07
PARHAS	most sensitive	0.1	4.940 ± 0.424	5.097 ± 0.424	5.860 ± 0.613	-0.031	-0.155	-1.23
PUQYAC	random middle	0.1	0.897 ± 0.044	0.853 ± 0.078	0.881 ± 0.064	0.05	-0.006	-0.07
QONQEQ	most sensitive	0.1	1.260 ± 0.241	1.272 ± 0.201	1.291 ± 0.239	-0.009	-0.02	-0.09
SENWEP	most sensitive	0.1	0.610 ± 0.176	0.552 ± 0.154	0.581 ± 0.154	0.1	0.001	0.0
SENWIT	most sensitive	0.1	0.177 ± 0.088	0.180 ± 0.094	0.172 ± 0.085	-0.018	0.034	0.06
TASXIW	least sensitive	0.1	0.844 ± 0.158	0.821 ± 0.151	0.837 ± 0.153	0.028	-0.005	-0.02
UXUZAP	least sensitive	0.1	2.283 ± 0.164	2.287 ± 0.170	2.319 ± 0.173	-0.002	-0.015	-0.16
VICYUD	random middle	0.1	3.159 ± 0.189	3.136 ± 0.213	3.212 ± 0.206	0.007	-0.02	-0.26
WEBREC	random middle	0.1	2.462 ± 0.210	2.466 ± 0.148	2.574 ± 0.132	-0.002	-0.044	-0.6
WEMNAF	random middle	0.1	2.483 ± 0.127	2.455 ± 0.099	2.628 ± 0.150	0.012	-0.062	-0.93
XEDLUN	random middle	0.1	6.352 ± 0.266	6.425 ± 0.239	6.448 ± 0.209	-0.011	-0.009	-0.22
XINWUO	most sensitive	0.1	0.132 ± 0.058	0.129 ± 0.058	0.127 ± 0.056	0.024	0.028	0.05
ZnBDC	random middle	0.1	2.755 ± 0.263	2.781 ± 0.235	2.757 ± 0.228	-0.009	0.004	0.04
BENXUP	least sensitive	1	3.044 ± 0.093	3.124 ± 0.057	3.059 ± 0.067	-0.026	0.008	0.29
BUVXOG	random middle	1	1.405 ± 0.177	1.275 ± 0.167	1.348 ± 0.169	0.097	-0.006	-0.04
CUYQIX	most sensitive	1	2.613 ± 0.027	2.274 ± 0.063	2.981 ± 0.089	0.139	-0.199	-5.6
EGELUY01	most sensitive	1	8.001 ± 0.345	8.002 ± 0.344	8.081 ± 0.298	-0.0	-0.01	-0.21
EHALOP	most sensitive	1	8.335 ± 0.270	8.273 ± 0.297	8.370 ± 0.300	0.007	-0.008	-0.18
FIPWUY	least sensitive	1	3.647 ± 0.085	3.783 ± 0.042	3.652 ± 0.067	-0.037	0.017	0.77
KAXQIL	random middle	1	1.571 ± 0.034	1.615 ± 0.032	1.640 ± 0.050	-0.028	-0.029	-0.86
LEGGEK	least sensitive	1	3.365 ± 0.066	3.522 ± 0.028	3.623 ± 0.083	-0.046	-0.051	-1.99
LOWPUI	least sensitive	1	5.186 ± 0.169	5.329 ± 0.209	5.108 ± 0.168	-0.027	0.029	0.7
LURGEL	most sensitive	1	3.225 ± 0.377	3.435 ± 0.424	3.284 ± 0.410	-0.063	0.014	0.09
MAZSUD	least sensitive	1	4.563 ± 0.259	4.648 ± 0.246	4.574 ± 0.244	-0.018	0.007	0.1
MEJQID	least sensitive	1	4.376 ± 0.181	4.387 ± 0.130	4.349 ± 0.158	-0.003	0.008	0.17
MEJQOJ	least sensitive	1	4.274 ± 0.203	4.424 ± 0.194	4.358 ± 0.155	-0.034	-0.002	-0.04
NAQLAT	least sensitive	1	4.272 ± 0.103	4.267 ± 0.070	4.353 ± 0.113	0.001	-0.019	-0.65
NARTUX	most sensitive	1	1.338 ± 0.162	1.597 ± 0.102	2.573 ± 0.097	-0.177	-0.561	-8.14
NISCOJ	random middle	1	2.354 ± 0.152	2.321 ± 0.122	2.391 ± 0.103	0.014	-0.023	-0.38
OGAGIN	random middle	1	2.905 ± 0.142	2.838 ± 0.170	3.037 ± 0.117	0.023	-0.056	-1.03
PARHAS	most sensitive	1	14.456 ± 0.445	14.323 ± 0.397	14.402 ± 0.416	0.009	-0.001	-0.02
PUQYAC	random middle	1	1.264 ± 0.047	1.292 ± 0.067	1.292 ± 0.054	-0.022	-0.01	-0.2
QONQEQ	most sensitive	1	8.127 ± 0.337	7.846 ± 0.298	8.011 ± 0.285	0.035	-0.003	-0.07
SENWEP	most sensitive	1	4.053 ± 0.298	4.214 ± 0.283	4.161 ± 0.311	-0.039	-0.007	-0.07
SENWIT	most sensitive	1	1.987 ± 0.328	1.957 ± 0.330	1.966 ± 0.311	0.015	0.003	0.02
TASXIW	least sensitive	1	5.735 ± 0.279	5.796 ± 0.286	5.707 ± 0.249	-0.01	0.01	0.18
UXUZAP	least sensitive	1	4.240 ± 0.121	4.367 ± 0.205	4.296 ± 0.160	-0.03	0.002	0.04
VICYUD	random middle	1	6.226 ± 0.203	6.328 ± 0.150	6.313 ± 0.180	-0.016	-0.006	-0.16
WEBREC	random middle	1	3.944 ± 0.066	4.027 ± 0.087	3.997 ± 0.071	-0.021	-0.003	-0.12
WEMNAF	random middle	1	3.835 ± 0.052	3.958 ± 0.139	3.902 ± 0.068	-0.032	-0.001	-0.05
XEDLUN	random middle	1	8.071 ± 0.171	8.092 ± 0.194	8.224 ± 0.160	-0.003	-0.018	-0.69
XINWUO	most sensitive	1	1.294 ± 0.183	1.327 ± 0.215	1.304 ± 0.183	-0.025	0.005	0.03
ZnBDC	random middle	1	5.465 ± 0.211	5.349 ± 0.150	5.441 ± 0.164	0.021	-0.006	-0.16

Table S12

Long-run (40,000-cycle) check of the conditions that disagreed with CRAFTED by more than 2σ after 10,000 cycles, and of the controls.

MOF	P / bar	Kind	10k mean / mol kg^{-1}	CRAFTED / mol kg^{-1}	z (10k)	Cycles	Long-run MSER mean / mol kg^{-1}	z (long run)
NARTUX	0.1	discrepant	1.249	1.876	-5.8	40000	1.635	-1.7
SENWEP	0.1	control	0.581	0.581	0.0	40000	0.599	0.08
EHALOP	0.1	control	1.313	1.315	-0.01	40000	1.303	-0.03
CUYQIX	1	discrepant	2.444	2.981	-5.6	40000	2.97	-0.1
NARTUX	1	discrepant	1.468	2.573	-8.14	40000	2.255	-1.52
SENWIT	1	control	1.972	1.966	0.02	40000	1.971	0.01
PARHAS	1	control	14.389	14.402	-0.02	40000	14.503	0.16

Table S13

Factor effects measured with RASPA3 on the 30 sampled MOFs, with the CRAFTED variance components and protocol effects at the same condition.

Factor	Condition	n	Mean Δ ln u	sd across MOFs	Run-to-run sd	Median reported relative uSD	Inside 2σ	Spearman |effect| vs sensitivity	Note
Replicate seed (seed 2 vs 1)	CO_2, 0.1 bar	30	0.0013	0.059	0.041	0.0855			sd of ln(u1)-ln(u2) / sqrt2 = true repeatability
Code (RASPA3 vs RASPA2)	CO_2, 0.1 bar	30	-0.0311	0.084			29.0	0.228	mean = code bias; sd = MOF-specific code effect (compare with CRAFTED interaction sd)
Replicate seed (seed 2 vs 1)	CO_2, 1 bar	30	-0.0098	0.0513	0.0363	0.0382			sd of ln(u1)-ln(u2) / sqrt2 = true repeatability
Code (RASPA3 vs RASPA2)	CO_2, 1 bar	30	-0.0306	0.1077			28.0	-0.068	mean = code bias; sd = MOF-specific code effect (compare with CRAFTED interaction sd)
Sampling (40,000 vs 10,000 cycles)	N_2, 1 bar	30	0.009	0.0277		0.2246	30.0		reported uSD shrinks by x1.01 (ideal 0.5); |z| vs CRAFTED 10k: 0.05 -> 40k: 0.02
CO_2 model (EPM2 vs TraPPE)	CO_2, 0.1 bar	30	-0.0556	0.0842			29.0	0.133	mean = factor main effect; sd = MOF-specific part; within_2sigma = MOFs where the change is inside MC noise
Cut-off (12.0 Å + tail vs 12.8 Å shifted)	CO_2, 0.1 bar	30	0.2277	0.1559			17.0	0.135	mean = factor main effect; sd = MOF-specific part; within_2sigma = MOFs where the change is inside MC noise
Long run, discrepant conditions (40,000 vs 10,000)	CO_2, 0.1/1 bar	3	0.298	0.1198			3.0		vs CRAFTED: mean ln diff -0.091 (10k runs: -0.389); |z| median 5.8 -> 1.5
Long run, control conditions (40,000 vs 10,000)	CO_2, 0.1/1 bar	4	0.0076	0.0162			4.0		vs CRAFTED: mean ln diff +0.008 (10k runs: +0.000); |z| median 0.0 -> 0.1
CRAFTED: protocol main effect	CO_2, 0.1 bar	632		0.3281					from Step 12
CRAFTED: material × protocol interaction	CO_2, 0.1 bar	632		0.4604					from Step 12
CRAFTED: Monte Carlo (reported)	CO_2, 0.1 bar	632		0.1574					from Step 12 (reported uSD)
CRAFTED charge scheme Qeq vs DDEC (UFF)		632	0.1265	0.7034					from Step 12 protocol_main_effects.csv
CRAFTED no charges vs DDEC (UFF)		632	-0.7507	0.6726					from Step 12 protocol_main_effects.csv
CRAFTED DREIDING vs UFF (DDEC)		632	-0.2399	0.4618					from Step 12 protocol_main_effects.csv

Table S14

Experimental anchor: measured CO_2 uptake from the NIST/ARPA-E database interpolated at 0.1 and 1 bar, against the CRAFTED protocol range at 298 K.

Material	CRAFTED CIF	NIST isotherm	Source DOI	T / K	Reported units	P / bar	Measured / mol kg^{-1}	UFF + DDEC / mol kg^{-1}	Protocol min	Protocol median	Protocol max	ln(measured/reference)	Inside range
IRMOF-1 (MOF-5)	IRMOF-1	10.1021ar500272n.Isotherm19	10.1021/ar500272n	298	cm3(STP)/cm3 vs bar	1	0.818	0.83	0.717	0.911	1.609	-0.014	yes
IRMOF-1 (MOF-5)	IRMOF-1	10.1016j.seppur.2018.01.013.Isotherm11	10.1016/j.seppur.2018.01.013	297	mmol/g vs bar	1	0.834	0.83	0.717	0.911	1.609	0.004	yes
ZIF-8	ZIF-8	10.1021acs.jced.1c00900.Isotherm8	10.1021/acs.jced.1c00900	298	mmol/g vs bar	1	0.68	1.612	0.872	1.27	1.852	-0.863	no
ZIF-8	ZIF-8	10.1007s109040140131z.Isotherm15	10.1007/s10904-014-0131-z	298	mmol/g vs bar	0.1	0.078	0.149	0.084	0.122	0.177	-0.653	no
ZIF-8	ZIF-8	10.1007s109040140131z.Isotherm15	10.1007/s10904-014-0131-z	298	mmol/g vs bar	1	0.962	1.612	0.872	1.27	1.852	-0.516	yes
ZIF-90	ZIF-90	10.1007s109040140131z.Isotherm14	10.1007/s10904-014-0131-z	298	mmol/g vs bar	0.1	0.216	0.273	0.083	0.186	0.724	-0.234	yes
ZIF-90	ZIF-90	10.1007s109040140131z.Isotherm14	10.1007/s10904-014-0131-z	298	mmol/g vs bar	1	2.297	2.882	0.835	1.924	5.548	-0.227	yes
Zn-MOF-74	ZnMOF-74	10.1021acs.jpcc.7b08239.Isotherm10	10.1021/acs.jpcc.7b08239	298	mmol/g vs bar	0.1	0.993	0.256	0.118	0.238	0.643	1.356	no
MIL-53(Al)	SABVUN	10.1016j.micromeso.2015.07.013.Isotherm15	10.1016/j.micromeso.2015.07.013	298	mmol/g vs bar	1	3.412	7.677	5.379	7.682	19.221	-0.811	no
NOTT-300	WAYMEQ	10.1007s10450020002829.Isotherm10	10.1007/s10450-020-00282-9	298	mmol/g vs bar	0.1	0.434	2.56	0.732	1.435	5.617	-1.774	no
DMOF-1	ZnBDCdabco	10.1016j.micromeso.2014.09.038.Isotherm7	10.1016/j.micromeso.2014.09.038	298	mmol/g vs bar	0.1	0.153	0.335	0.156	0.256	0.385	-0.781	no
DMOF-1	ZnBDCdabco	10.1016j.micromeso.2014.09.038.Isotherm7	10.1016/j.micromeso.2014.09.038	298	mmol/g vs bar	1	1.739	4.25	1.688	2.995	5.052	-0.894	yes

Table S15

Scatter between laboratories and between protocols for the materials measured by more than one group.

Material	P / bar	Laboratories	sd between laboratories (ln)	sd between protocols (ln)
IRMOF-1 (MOF-5)	1	2	0.013	0.244
ZIF-8	1	2	0.245	0.309

Table S16

Structure source: reference-protocol uptake (UFF + DDEC, 298 K) across the CoRE MOF 2014 CIFs of the same composition.

Material	P / bar	CIFs	Min / mol kg^{-1}	Max / mol kg^{-1}	ln(max/min)	Other CoRE MOF 2014 CIFs
IRMOF-1 (MOF-5)	0.1	21	0.076	0.577	2.032	SAHYOQ03, SAHYOQ04, SAHYOQ05, EDUSIF, HIFTOG, HIFTOG01, HIFTOG02, LAWGEW, LAWGIA, LAWGOG, LAWGUM, MIBQAR, MIBQAR16, MIBQAR18, PEVQEO, PEVQIS, PEVQOY, UNIGEE, VAZTOG, VUSKAW,

						VUSKEA
IRMOF-1 (MOF-5)	1	22	0.768	3.668	1.563	SAHYOQ03, SAHYOQ04, SAHYOQ05, EDUSIF, HIFTOG, HIFTOG01, HIFTOG02, LAWGEW, LAWGIA, LAWGOG, LAWGUM, MIBQAR, MIBQAR16, MIBQAR18, PEVQEO, PEVQIS, PEVQOY, UNIGEE, VAZTOG, VUSKAW, VUSKEA
MIL-53(Al)	0.1	8	0.968	1.930	0.690	EGELUY01, EHALOP, QONQEQ, WAYMIU, WAYMOA, SABVOH01, HAFQOW, HAFQUC
MIL-53(Al)	1	9	6.824	8.820	0.256	EGELUY01, EHALOP, QONQEQ, WAYMIU, WAYMOA, SABVOH01, HAFQOW, HAFQUC
ZIF-8	0.1	4	0.137	0.176	0.247	MECWEX, OFERUN04, TUDJOS
ZIF-8	1	4	1.427	1.612	0.122	MECWEX, OFERUN04, TUDJOS
Zn-MOF-74	0.1	2	0.256	0.817	1.161	Zn-DOBDC
Zn-MOF-74	1	1	5.654	5.654	0.000	Zn-DOBDC
